# Malignant peritoneal mesotheliomas of rats induced by multiwalled carbon nanotubes and amosite asbestos: transcriptome and epigenetic profiles

**DOI:** 10.1186/s12989-024-00565-x

**Published:** 2024-01-31

**Authors:** Stella Marie Reamon-Buettner, Susanne Rittinghausen, Annika Klauke, Andreas Hiemisch, Christina Ziemann

**Affiliations:** Fraunhofer Institute for Toxicology and Experimental Medicine (ITEM), Nikolai-Fuchs-Strasse 1, 30625 Hannover, Germany

**Keywords:** Mesothelioma, Multiwalled carbon nanotubes, Amosite asbestos, Genome-wide transcriptome analyses, DNA methylation, RNA methylation

## Abstract

**Background:**

Malignant mesothelioma is an aggressive cancer that often originates in the pleural and peritoneal mesothelium. Exposure to asbestos is a frequent cause. However, studies in rodents have shown that certain multiwalled carbon nanotubes (MWCNTs) can also induce malignant mesothelioma. The exact mechanisms are still unclear. To gain further insights into molecular pathways leading to carcinogenesis, we analyzed tumors in Wistar rats induced by intraperitoneal application of MWCNTs and amosite asbestos. Using transcriptomic and epigenetic approaches, we compared the tumors by inducer (MWCNTs or amosite asbestos) or by tumor type (sarcomatoid, epithelioid, or biphasic).

**Results:**

Genome-wide transcriptome datasets, whether grouped by inducer or tumor type, showed a high number of significant differentially expressed genes (DEGs) relative to control peritoneal tissues. Bioinformatic evaluations using Ingenuity Pathway Analysis (IPA) revealed that while the transcriptome datasets shared commonalities, they also showed differences in DEGs, regulated canonical pathways, and affected molecular functions. In all datasets, among highly- scoring predicted canonical pathways were *Phagosome Formation*, *IL8 Signaling*, *Integrin Signaling*, *RAC Signaling*, and *TREM1 Signaling.* Top-scoring activated molecular functions included *cell movement*, *invasion of cells*, *migration of cells*, *cell transformation*, and *metastasis*. Notably, we found many genes associated with malignant mesothelioma in humans, which showed similar expression changes in the rat tumor transcriptome datasets. Furthermore, RT-qPCR revealed downregulation of *Hrasls, Nr4a1, Fgfr4, and Ret* or upregulation of *Rnd3* and *Gadd45b* in all or most of the 36 tumors analyzed. Bisulfite sequencing of *Hrasls, Nr4a1, Fgfr4, and Ret* revealed heterogeneity in DNA methylation of promoter regions. However, higher methylation percentages were observed in some tumors compared to control tissues. Lastly, global 5mC DNA, m6A RNA and 5mC RNA methylation levels were also higher in tumors than in control tissues.

**Conclusions:**

Our findings may help better understand how exposure to MWCNTs can lead to carcinogenesis. This information is valuable for risk assessment and in the development of safe-by-design strategies.

**Supplementary Information:**

The online version contains supplementary material available at 10.1186/s12989-024-00565-x.

## Background

Malignant mesothelioma is a rare but aggressive cancer that develops in mesothelial cells. These cells are found in the serosal membranes of the pleura, peritoneum, pericardium, and tunica vaginalis testes. Mesothelial cells play an important role in maintaining the integrity and function of serosal membranes. They produce various mediators in response to external signals, which help regulate inflammatory, immune, and tissue repair responses [1]. Mesotheliomas are usually designated based on the location of the affected membrane. The most common type is pleural mesothelioma affecting the visceral pleura, then followed by peritoneal mesothelioma affecting the peritoneum. Due to their frequency, most studies were undertaken on pleural mesotheliomas. Mesotheliomas are also classified according to World Health Organization (WHO) criteria into three subtypes based on histopathology: epithelioid, sarcomatoid, and biphasic/mixed. The sarcomatoid and biphasic types are associated with a poorer prognosis than the epithelioid type [2].

The development of malignant mesothelioma is usually associated with asbestos exposure. It may take up to 40 years from the time of exposure to asbestos for malignancy to manifest. This process may involve diverse mechanisms and signaling pathways, depending on how asbestos fibers interact with mesothelial cells [3]. Other properties may also play a role in the development of asbestos-induced mesothelioma. For example, a recent study has shown differences in the internal morphology of asbestos ferruginous bodies (AFBs), which are a key indicator of asbestos-induced malignant mesothelioma, between smokers and non-smokers [4]. Recent advances in high-throughput technologies have helped reveal molecular cues, including genomic and epigenetic factors and their interplay with the immune response, that are relevant to mesothelioma development [5, 6]. Additionally, preclinical models of mesothelioma have been useful in deciphering the disease’s pathogenesis and in developing new clinical interventions [7].

Carbon nanotubes (CNTs) are fiber-like nanomaterials with a wide range of promising applications in industry. They can be used in composite materials [8] or in biomedicine for drug delivery, cancer therapy, biosensors, imaging, and tissue engineering [9, 10]. Manufactured CNTs can be generally classified as single-walled (SWCNT), double-walled (DWCNT) or multiwalled (MWCNT) types [10, 11]. But owing to their fiber-like structure similar to asbestos, CNTs can induce disruptions on the genome and epigenome to cause diseases, such as cancer. For instance, exposure to CNTs triggered epigenetic changes in mouse model [12] and human bronchial epithelial cells [13–15]. Indeed, several studies have shown that certain MWCNTs can induce malignant mesothelioma in animal models. These studies administered MWCNTs by intraperitoneal injection in rats [16–18], intratracheal instillation in rats [19–21] or intrapleural injection in mice [22]. However, the exact mechanisms and signaling pathways that lead to malignancy after exposure to MWCNTs remain unclear. While asbestos and MWCNTs can perturb identical pathways leading to the induction of mesothelioma [22, 23], they may also induce distinct responses [24].

Previously, by using in *vitro* methods on primary human mesothelial cells, we showed that certain long and straight MWCNTs could induce characteristic markers of cellular senescence [25]. We observed inhibition of cell division, senescence-associated heterochromatin foci, senescence-associated distension of satellites, LMNB1 depletion, γH2A.X nuclear panstaining, and enlarged cells exhibiting senescence-associated β-galactosidase activity. In this present study, we aimed to gain further insights into mechanisms and signaling pathways that contribute to the development of mesothelioma from exposure to MWCNTs. We used various approaches to analyze the transcriptome and epigenome profiles of tumors induced by intraperitoneal application of three different MWCNTs or amosite asbestos in Wistar rats. These tumors were part of an in vivo carcinogenicity study to identify the potential carcinogenic effects of tailor-made, non-functionalized MWCNTs [17].

We compared the tumors based on their inducers (MWCNTs or amosite asbestos) and their tumor types (sarcomatoid, epithelioid, or biphasic). Despite some differences, the tumors shared common features in their transcriptome and epigenome, regardless of their inducer or type. Notably, we identified 38 differentially expressed genes (DEGs), implicated in mesothelioma or mesothelioma formation, that showed consistent expression changes across all transcriptome datasets. Interestingly, 17 of these DEGs were similarly expressed after comparison with publicly available human malignant pleural mesothelioma datasets. Our findings may help in a better understanding of MWCNT-induced carcinogenesis and may provide valuable insights for risk assessment and in the development of safe-by-design strategies.

## Results

### Genome-wide transcriptome analysis shows similarities and differences in tumors induced by MWCNTs and amosite asbestos

**Fig. 1:**
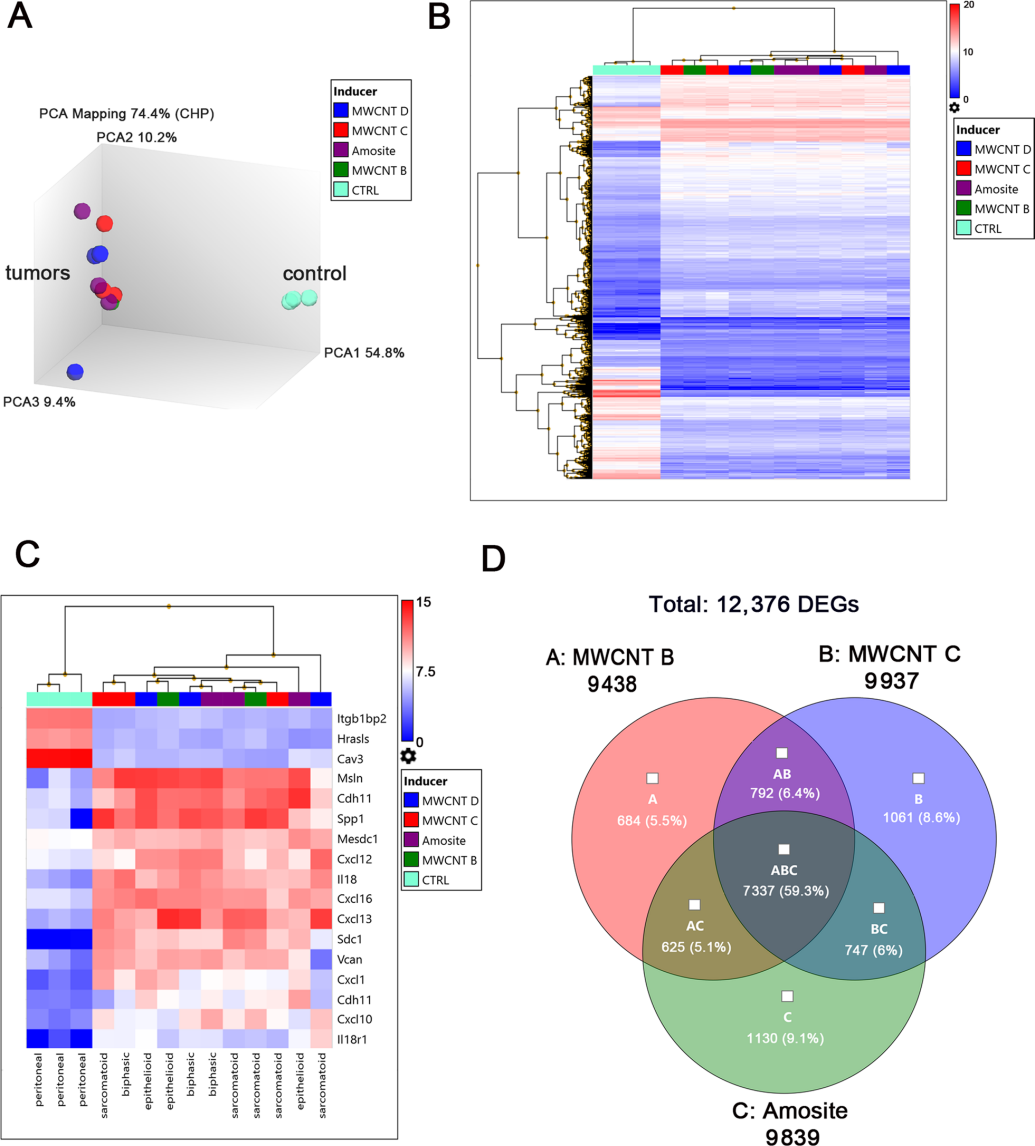
Transcriptome profiling of tumors induced by MWCNTs and amosite asbestos. **A** Principal component analysis (PCA) shows clear separation of tumors from the control peritoneal tissues. **B** Unsupervised hierarchical clustering analysis and heat map of 3062 filtered genes at fold change < − 2 or > 2, ANOVA *P* < 0.001, and FDR *P* < 0.001. **C** Hierarchical clustering and heat map on a few selected genes encoding for proteins such as mesothelin, osteopontin, caveolin, integrin, syndecan, chemokine, and interleukin. **D** Venn diagrams display the quantity of genes that are common and unique among the datasets of MWCNT B, MWCNT C, and amosite asbestos. The genes in the Venn diagrams were filtered using fold change < − 2 or > 2, ANOVA *P* < 0.05, and FDR *P* < 0.05. Quality control criteria and bioinformatics tools were according to Transcriptome Analysis Console (TAC 4.0.2, Thermo Fisher Scientific)

We carried out a genome-wide transcriptome analysis using Affymetrix microarrays on 11 tumors induced by MWCNTs or amosite asbestos, and 3 control peritoneal tissues. The principal component analysis (PCA) showed that the transcriptomes of the tumors grouped together, distinct from the control tissues (Fig. 1A). Hierarchical cluster analysis (unsupervised or on a few selected genes), also showed distinct clustering of tumor transcriptomes from those of control tissues (Fig. 1B, C). Visualization of the transcriptome data by Venn diagrams revealed both common and unique differentially expressed genes (DEGs) in the tumors induced by MWCNTs and amosite asbestos (Fig. 1D).

For further analysis of differential comparison of expressed genes between the tumors and control peritoneal tissues, we classified the tumors according to inducer or tumor type. The total number of DEGs in the tumors did not vary much between inducers (Table 1). On average, we found 9806 DEGs, of these 68.6% showed increased expression (upregulated), while 31.4% showed decreased expression (downregulated). When comparing results by tumor type, we found similar results. On average, there were 10,612 DEGs, of which 69.4% were upregulated and 30.6% were downregulated. The transcriptome data, when visualized through Venn diagrams, also showed both common and unique DEGs in the different tumor types (see Additional file 5: Figure S1A). From the total of 13,383 DEGs, 7866 (57.9%) were common to all three types of mesotheliomas. Sarcomatoid tumors had the highest number of unique DEGs with 1301 (9.6%), followed by epithelioid tumors with 1045 DEGs (7.7%), and biphasic tumors with 848 DEGs (6.2%). Furthermore, shown in Additional file 5: Figure S1B–D are the sample signals obtained for the genes encoding for forehead box M1 (Foxm1), mesothelin (Msln), and secreted phosphoprotein 1/osteopontin (Spp1) in the different tumor types and control peritoneal tissues. Sample signals, which represent the measured intensities of gene expression for each sample, were higher in all the tumor types than in the control peritoneal tissues.

**Table 1:** Summary of genome-wide transcriptome analysis in tumors by inducer or tumor type relative to control peritoneal tissues

Material	Total differentially expressed genes (DEGs)*	Upregulated genes	Downregulated genes
MWCNT B	9438	6467 (68.52%)	2971 (31.48%)
MWCNT C	9937	6747 (67.90%)	3190 (32.10%)
MWCNT D	10,011	6856 (68.48%)	3155 (31.52%)
Amosite	9839	6856 (69.56%)	2995 (30.44%)
Sarcomatoid	10,649	7170 (67.33%)	3479 (32.67%)
Epithelioid	10,587	7526 (71.09%)	3061 (28.91%)
Biphasic	10,602	7411 (69.90%)	3191 (30.10%)

^*^DEGs passing the filter: fold change < -2 or > 2; ANOVA *P* < 0.05; and FDR *P* < 0.05

**Fig. 2:**
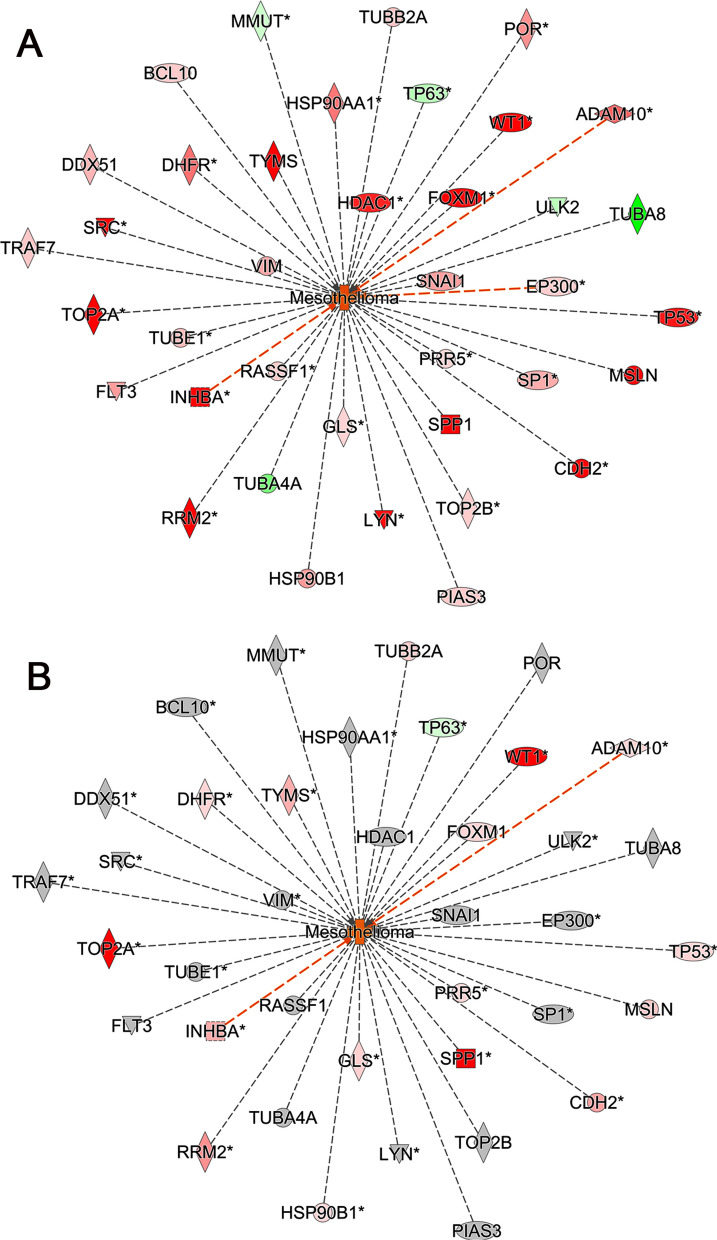
Genes implicated in mesothelioma or mesothelioma formation. **A** Thirty-eight differentially expressed genes (DEGs) that showed consistent expression changes across the rat transcriptome datasets, regardless of inducer or tumor type. Overlay gene expressions (fold changes) represent those of MWCNT C. **B** The same set of genes is overlaid with gene expressions (fold changes) from the dataset GSE51024, which pertains to human malignant pleural mesotheliomas. Genes were filtered by fold change < − 1.5 or > 1.5, ANOVA *P* < 0.05, and FDR *P* < 0.05. Red (upregulated), green (downregulated), gray (did not meet at least one filter), orange (predicted activation)

To better understand the biological significance of the microarray data, we applied several bioinformatic approaches using Ingenuity Pathway Analysis (IPA). First, we searched for DEGs in the transcriptome datasets, which are implicated in mesothelioma or mesothelioma formation. In our analysis, we identified 38 DEGs that showed consistent changes in expression across all transcriptome datasets. This was true regardless of whether the datasets were grouped by inducer or by tumor type (Fig. 2A, Additional file 1: Table S1). For instance, *mesothelin* (*Msln*), *osteopontin* (*Spp1*), *forkhead box MI* (*Foxm1*), *thymidylate synthetase* (*Tyms*), and *Wilms' tumor suppressor gene* (*Wt1*), were among those upregulated genes. Notably, *ADAM metallopeptidase domain 10* (*Adam10*) and *inhibin subunit beta A* (*Inhba*) were also upregulated, and their upregulation was predicted to lead to the activation of mesothelioma formation (Fig. 2A).

To determine if the findings in rat mesotheliomas could be translated to human malignant mesotheliomas, we searched the Gene Expression Omnibus (GEO) database for relevant and publicly available gene expression datasets. However, we did not find any transcriptome datasets on human peritoneal mesotheliomas, specifically that have been obtained using Affymetrix microarrays and for which raw data are available. One possible explanation is that human pleural mesothelioma is more prevalent than peritoneal mesothelioma, resulting in more research being conducted on the former. Consequently, findings from pleural mesothelioma studies are often extrapolated to peritoneal mesothelioma [26]. After applying the same bioinformatics tools, we compared our rat transcriptome datasets with that of GSE51024 [27], which comprised 55 human malignant pleural mesotheliomas (MPM), and 41 lung parenchyma samples. Interestingly, we found that 17 of the 38 DEGs in the rat datasets were similarly expressed in the human MPM dataset (Fig. 2B, Additional file 2: Table S2). Furthermore, to determine whether similar results could be obtained using a different type of human tumor, we compared the rat transcriptome datasets with that of GSE149507 [28]. This dataset consisted of 18 pairs of small cell lung cancer (SCLC) tumors and adjacent lung tissues. In contrast to the human MPM dataset, where 17 of the 38 DEGs showed similar expression, only 9 of the 38 DEGs showed similar expression in the human SCLC tumor dataset (Additional file 6: Figure S2). These 9 DEGs, which were also similarly expressed in the human MPM dataset, included genes such as *Spp1* and *Foxm1* but not *Msln, Adam10*, and *Inhba*.

**Fig. 3:**
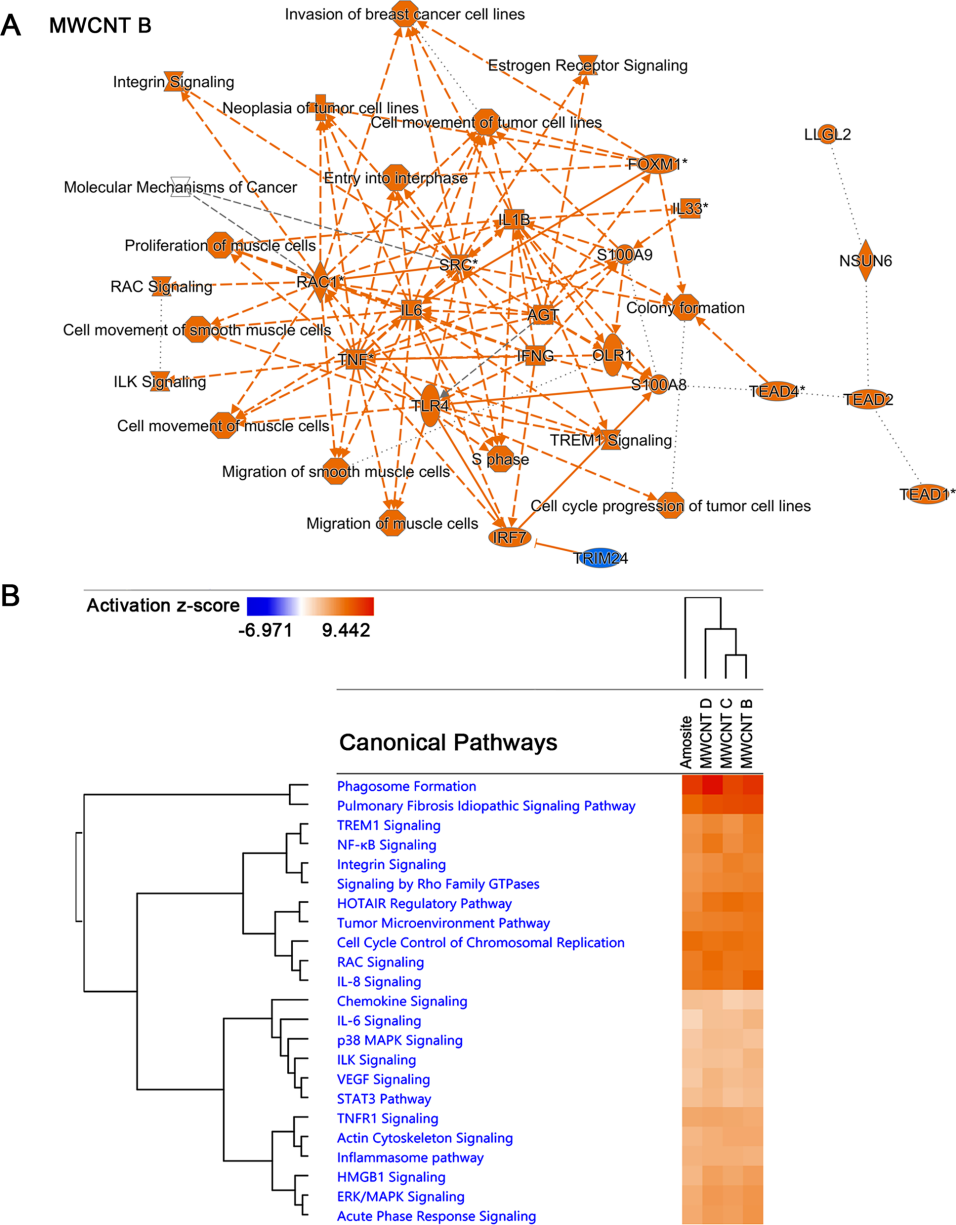
Bioinformatic analyses of tumor transcriptomes induced by MWCNTs and amosite asbestos using Ingenuity Pathway Analysis (IPA). **A** Graphical summary obtained by *Core Analysis* of the MWCNT B transcriptome dataset. The summary shows a relationship of a subset of the most significant entities predicted in the analysis, which include canonical pathways, upstream regulators, diseases, and biological functions. IPA graph legends: orange (activation); blue (inhibition); solid line (direct interaction); dashed line (indirect interaction); dotted line (inferred edge); for signaling pathways: an arrow pointing from A to B signifies that A causes B to be activated. **B** After conducting *Comparative Analysis*, shown are highly-scoring predicted canonical pathways in the datasets as classified by inducers. Heat maps depict z-scores, in which orange (positive z-score) represents activation. The canonical pathways are sorted by hierarchical clustering and z-scores. The column clusters represent the inducers

Second, we carried out *Core* and *Comparative Analysis* to identify biological changes across different transcriptome datasets of tumors. A graphical summary presented as a network of the key findings from the *Core Analysis* of the MWCNT B dataset is shown in Fig. 3A. This summary shows a relationship of a subset of the most significant entities predicted by the analysis, including canonical pathways, upstream regulators, diseases, and biological functions. In the transcriptome of tumors induced by MWCNT B, cytokines such as tumor necrosis factor (Tnf), interleukin 6 (Il6), interleukin 1 beta (Il1b), interleukin 33 (Il33), and interferon gamma (Ifng) were represented to be activated. Significant canonical pathways included *Molecular Mechanisms of Cancer*, *ILK Signaling*, *Integrin Signaling*, *RAC Signaling*, and *TREM1 Signaling*. Additionally, the molecular function *colony formation* was shown to be activated through the activation of forkhead box MI (Foxm1), SRC proto-oncogene (Src), and TEA domain transcription factor 4 (Tead 4).

**Fig. 4:**
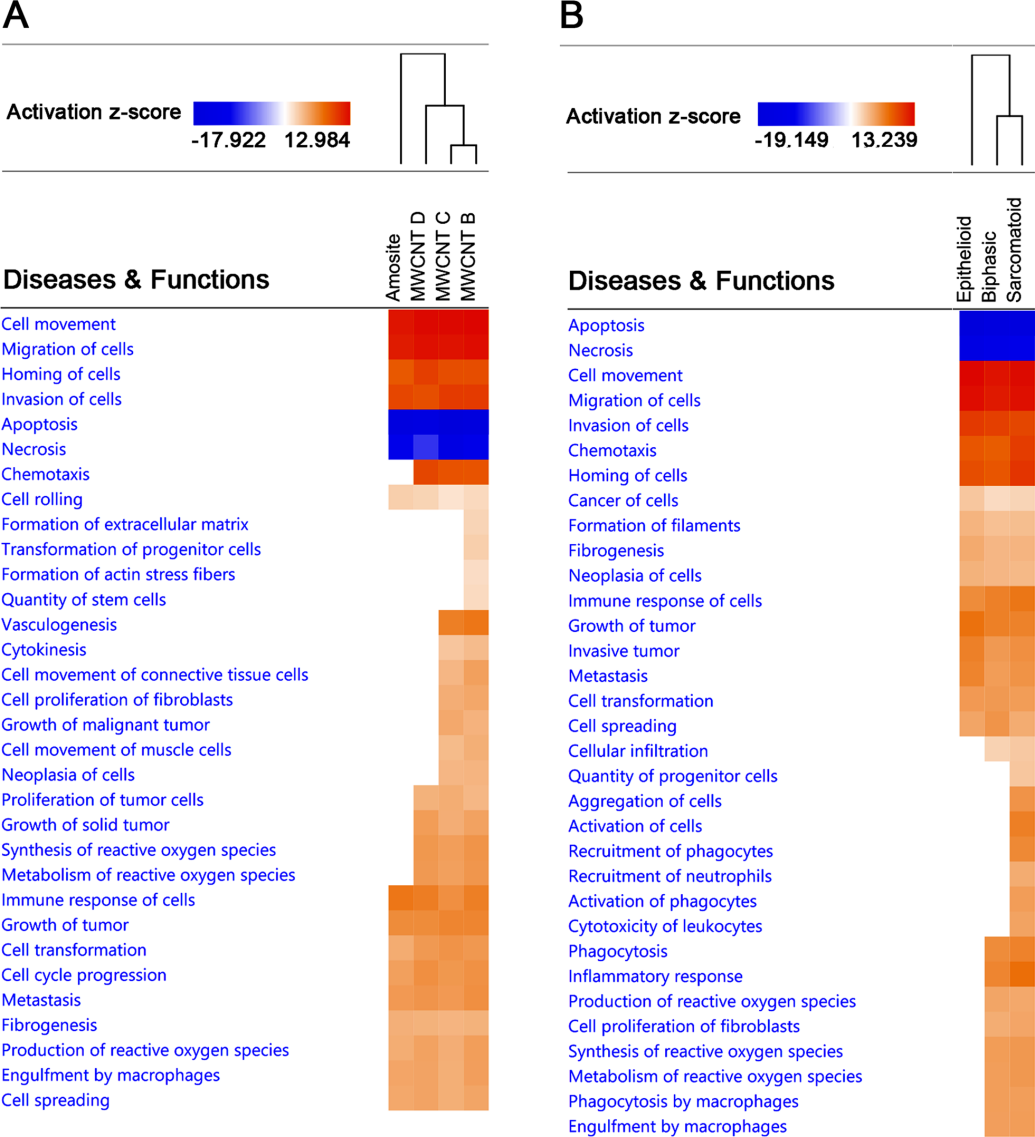
*Comparative Analysis* of diseases and molecular functions of transcriptomes of tumors induced by MWCNTs and amosite asbestos. **A** Datasets are classified by inducers. **B** Datasets are classified by type of mesothelioma. Heat maps depict z-scores, in which orange (positive z-score) represents activation, while blue (negative z-score) represents inhibition. No color means the function being analyzed is not involved in the dataset. The column clusters represent the inducers or type of mesothelioma. Data analysis and heat map generation were performed using Ingenuity Pathways Analysis (IPA)

After performing *Comparative Analysis* of transcriptome datasets grouped by inducers, we found that among the highly scoring common significant canonical pathways were *Phagosome Formation*, *IL8 Signaling*, *Integrin Signaling*, *RAC Signaling*, and *TREM1 Signaling* (Fig. 3B). Furthermore, when we carried out hierarchical clustering of these highly scoring canonical pathways and their z-scores, we observed that MWCNTs clustered differently from amosite asbestos. Characteristics of cancer cells and metastasis were among the highly scored molecular functions predicted to be regulated, regardless of classification by inducer (Fig. 4A) or tumor type (Fig. 4B). For instance, functions such as *cell movement*, *migration, homing, invasion,* and *spreading* were activated, while *apoptosis* was inhibited. Certain functions, however, were more activated in the tumors induced by MWCNTs than amosite asbestos (Fig. 4A). Hierarchical clustering showed clustering that reflected the tumor induction potential of the MWCNTs, and amosite asbestos observed during the in vivo study in rats [17]. Hierarchical clustering also reflected that sarcomatoid tumors are more aggressive than biphasic and epithelioid tumors (Fig. 4B).

**Fig. 5:**
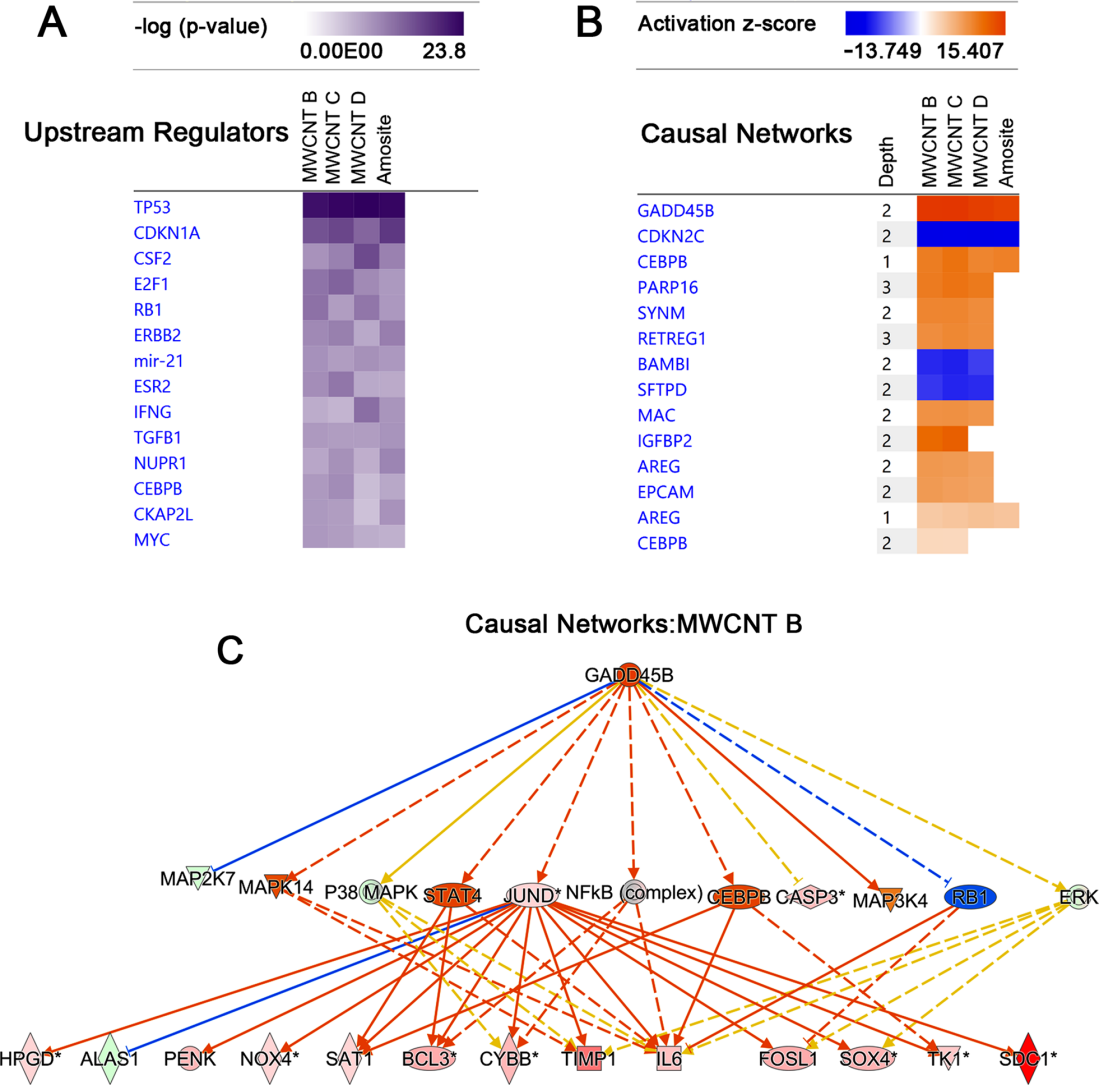
*Upstream Analysis* to identify novel master upstream regulators and causal networks in the transcriptome datasets of tumors induced by MWCNTs and amosite asbestos. **A** Highly scoring upstream regulators, ranked by *P*-values from right-tailed Fisher's exact test. **B** Predicted upstream regulators of causal networks. Heat map depicts z-scores, in which orange (positive z-score) represents activation, while blue (negative z-score) represents inhibition. No color means the pathway or function being analyzed is not involved in the dataset. C A causal network with Gadd45b as an upstream regulator with 11 intervening regulators to explain differential expression of 630 genes in the transcriptome dataset MWCNT B. Shown are 13 target genes directly regulated by Jund. Graph legends: orange (activation); blue (inhibition); solid line (direct interaction); dashed line (indirect interaction); upregulated (red), downregulated (green), gray (did not meet at least one filter). Data analysis and graph generation were done using Ingenuity Pathways Analysis (IPA)

Furthermore, we carried out *Upstream Analysis* to identify master regulators across the transcriptome datasets. The activation or inhibition of a particular upstream molecule would lead to the gene expression changes observed in the datasets. Shown in Fig. 5A are examples of highly significant upstream regulators. These include tumor suppressor protein p53 (Tp53), colony stimulating factor 2 (Csf2), and E2F transcription factor 1 (E2f2). All these regulators were also predicted to be activated. We also performed *Causal Network Analysis* to further determine higher-level master regulators, which could have also influenced the gene expression changes in the transcriptome datasets of the tumors. In the analysis, Gadd45b was identified as a top master regulator. It was predicted to be activated with a z-score of 15. 299, 15.407, 14.769 and 14.275 for MWCNT B, MWCNT C, MWCNT D, and amosite asbestos datasets, respectively. The causal network with Gadd45b as a master regulator involved a depth of 2 and 11 other intervening regulators, that could helped explain the up- and downregulation of 630 genes in MWCNT B dataset (Fig. 5B, C, Additional file 3: Table S3). The canonical pathways that shared the highest overlap with the regulators in this causal network were Senescence Pathway (8 of 11) and Hepatic Fibrosis Signaling Pathway (7 of 11).

### RT-qPCR also reveals gene expression changes in cancer-related genes in tumors induced by MWCNTs and amosite asbestos

To confirm the accuracy and robustness of our microarray data from the genome-wide transcriptome analysis, we performed RT-qPCR on a few selected genes that have been implicated in carcinogenesis. These genes were found be either downregulated: *Hras-like suppressor (Hrasls), Nuclear receptor subfamily 4, group A, member 1 (Nr4a1), Fibroblast growth factor receptor 4 (Fgfr4*), and *Ret-proto-oncogene (Ret),* or upregulated: *Rho family GTPase3* (*Rnd3/RhoE*) and *Growth arrest and DNA damage inducible beta (Gadd45b),* by the microarray analysis. We analyzed 4 control peritoneal tissues and 36 tumors induced by the three MWCNTs or amosite asbestos. The RT-qPCR results corroborated the microarray data and provided further insight into the role of these genes in carcinogenesis. These results will be described next.

**Fig. 6:**
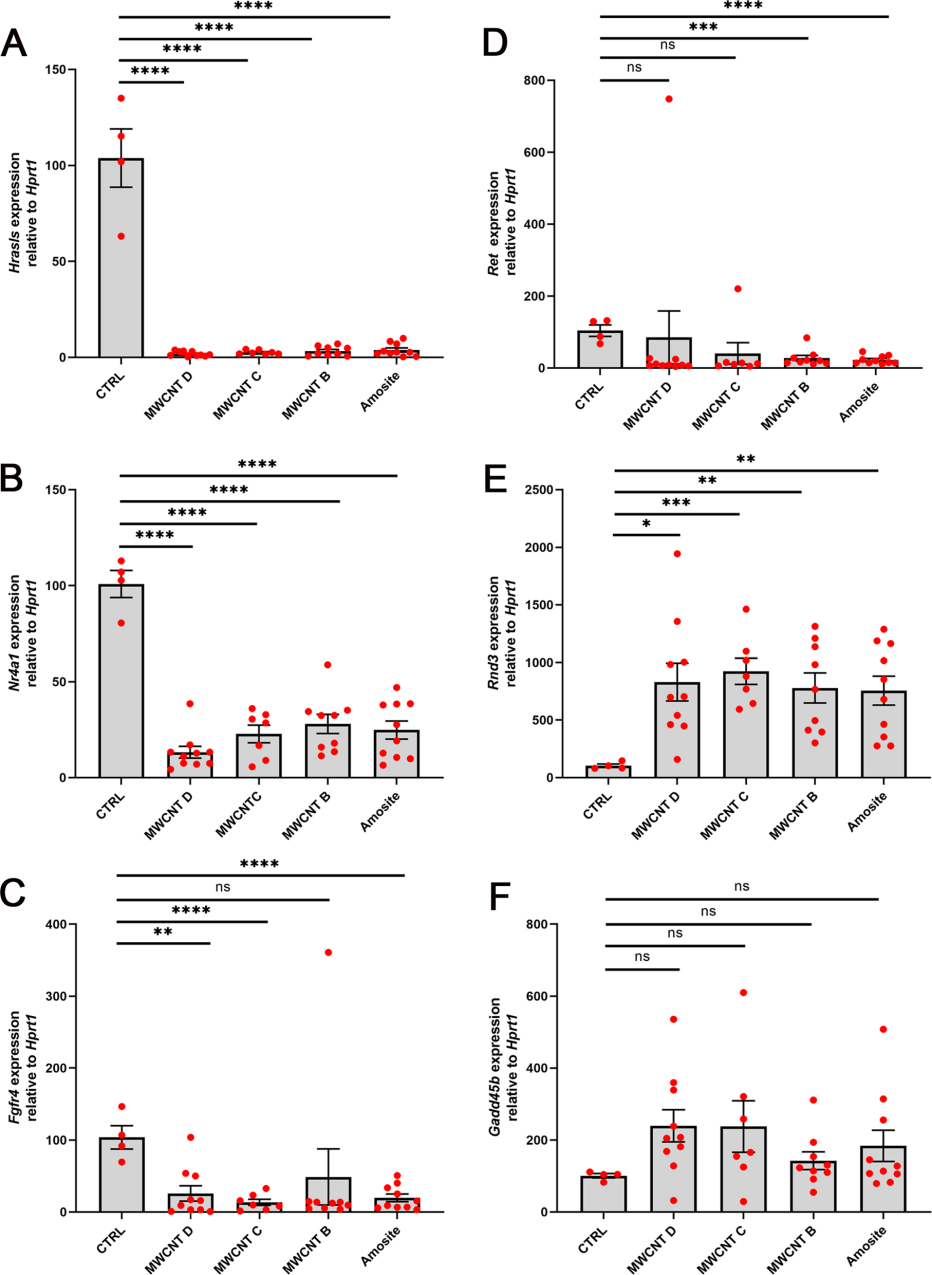
RT-qPCR results of selected cancer-associated genes in tumors induced by MWCNTs or amosite asbestos. As compared to the control peritoneal tissues, mRNA expression was downregulated in *Hrasls*, *Nr4a1*, *Fgfr4* and *Ret* (**A**–**D**) or upregulated in *Rnd3* and *Gadd45b* (**E**, **F**) in all or most of 36 tumors. Scatter dot plots and statistical results were obtained using GraphPad Prism 9. Statistical significance was determined at *P* < 0.05, *t*–test for unpaired values, two-tailed. Bar depicts mean and standard error of the mean (SEM). Asterisks depict statistical significance at **** (*P* < 0.0001), *** *(P* < 0. 001), **(*P* < 0. 01), * (*P* < 0. 05)

*Hrasls* is also known as *Phospholipase A and acyltransferase 1* (*Plaat1*) among other aliases and belongs to the *HRASLS (PLAAT)* family consisting of five members (1–5) in humans, and three (1,3, and 5) in mice and rats [29]. The enzymes encoded by these genes are important in diverse biological functions, including tumor suppression, and organelle degradation [30]. *Hrasls* was downregulated in 36 tumors relative to the 4 control tissues (Fig. 6A).

*Nr4a1* encodes an orphan nuclear receptor that functions as a ligand-independent transcription factor. In human cancer, NR4A1 has a conflicting role, acting both as a tumor suppressor and an oncogenic driver [31]. For instance, in certain samples of human breast cancer, NR4A1 gene and protein expressions were decreased to suggest a tumor suppressor role [32]. *Nr4a1* was downregulated in all 36 tumors relative to the 4 control tissues (Fig. 6B).

*Fgfr4* encodes one of the four tyrosine kinase receptors for fibroblast growth factors (FGFs). FGF and their receptors (known as FGF/FGFR signaling) regulate various cellular functions, including tumor progression [33]. In a wide range of tumors in patients, *FGFR4* is frequently overexpressed and exhibits sequence variations [34]. *Fgfr4* was downregulated in 94% (34 of 36) of the tumors relative to the 4 control tissues (Fig. 6C). This discrepancy may be accounted to other genetic and or epigenetic variations present in the rat tumors, as will be shown later.

*Ret,* which was generated by recombination between two unlinked DNA fragments, encodes a transmembrane receptor with tyrosine kinase (RTK) activity [35]. RET rearrangements have been detected in a variety of human cancers. *Ret* was downregulated in 92% (33 of 36) of the tumors relative to the 4 control tissues (Fig. 6D).

*Rnd3/RhoE* belongs to a family of genes, which encode small G proteins with important role in tumor initiation and progression [36]. Rho GTPases regulate proliferation and apoptosis, metabolism, senescence, and cancer cell stemness. Rnd3/RhoE has been implicated in cancer cell motility and its mRNA and protein expression can be upregulated by DNA damage-inducing stimuli [37]. *Rnd3* was upregulated in 36 tumors relative to the 4 control tissues (Fig. 6E).

*Gadd45b* is a member of the growth arrest DNA damage-inducible gene family. Members of this family have been implicated in many cellular functions such as DNA repair, cell cycle control, genotoxic stress response, and tumorigenesis [38]. In human cancer, *GADD45B* has been reported either as tumor suppressor gene or as oncogene [39]. *Gadd45b* was upregulated in 75% (27 of 36) of the tumors relative to the 4 control tissues (Fig. 6F).

### Tumors exhibit CpG and non-CpG DNA methylation in gene promoter regions

**Fig. 7:**
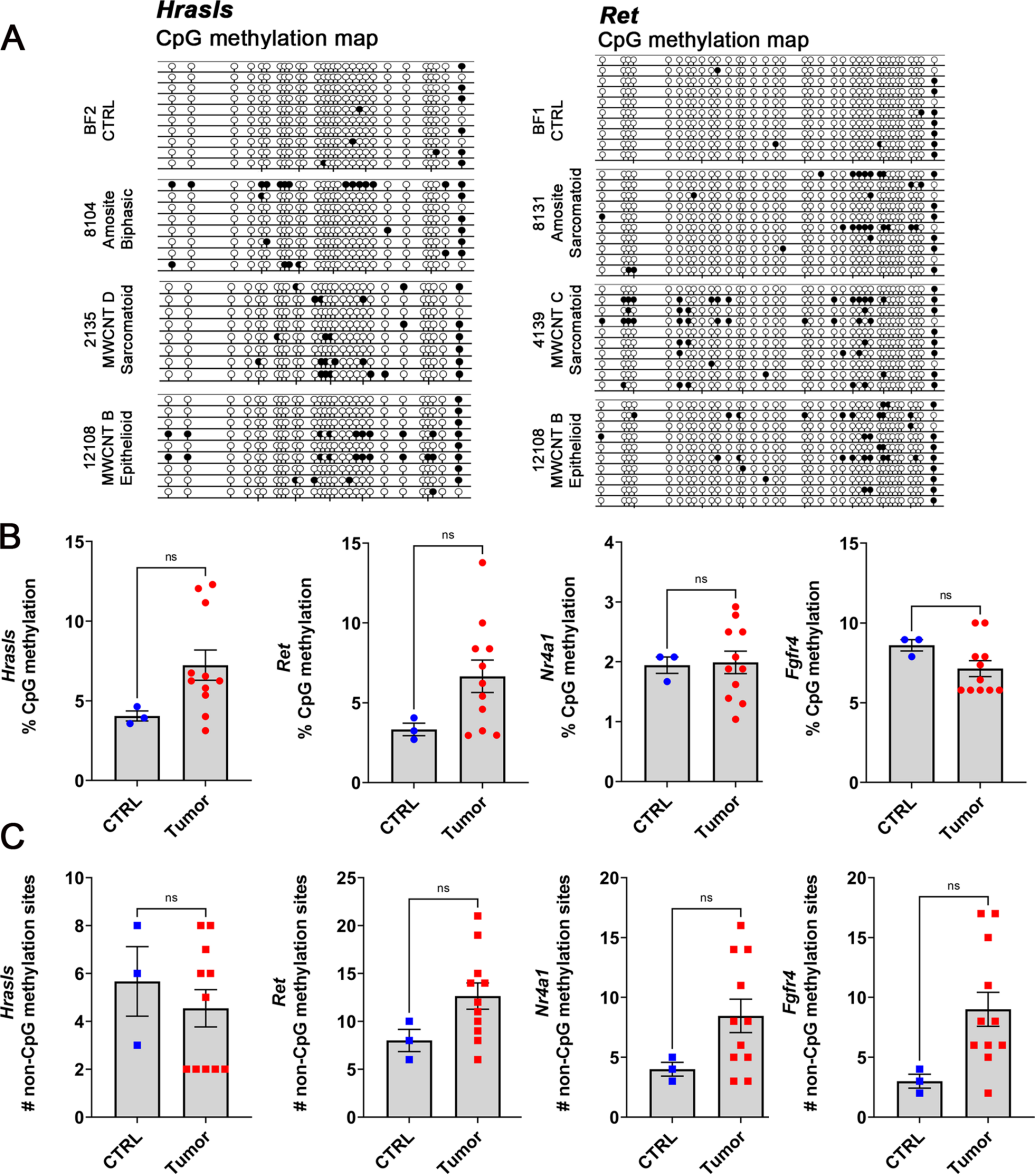
DNA methylation in the promoter regions of genes that are downregulated in tumors induced by MWCNTs or amosite asbestos. **A** Examples of CpG methylation maps for the *Hrasls* and *Ret* genes obtained after Sanger bisulfite sequencing. Shown are methylation patterns of 8–10 sequenced clones from both control and tumor tissues. Methylated CpGs (filled lollipops); Unmethylated CpGs (unfilled lollipops). **B**, **C** Percent CpG methylation and the number of non-CpG methylation sites in 3 control and 11 tumor tissues in *Hrasls, Ret*, *Nr4a1*, and *Fgfr4*. Scatter dot plots and statistical results were obtained using GraphPad Prism 9. Statistical significance was determined at *P* < 0.05, *t*–test for unpaired values, two-tailed. Bar depicts mean and standard error of the mean (SEM)

Our RT-qPCR analyses showed that the mRNA expression of *Hrasls, Ret*, *Nr4a1,* and *Fgfr4* was downregulated in all or most of the tumors we analyzed. To determine if DNA methylation, an epigenetic change, could be responsible for the repression of their transcription, we conducted bisulfite sequencing along the promoter regions of these genes in 11 tumors and 3 control peritoneal tissues. For each gene, we sequenced on average 8–10 clones from either tumor or control tissue, to identify methylated sites in the amplified fragments. For *Hrasls*, we analyzed a 431-bp fragment (28 CpGs, c.-376 to c.-46, relative to translation start site, ATG). Although overall percent CpG methylation between tumor and control tissues was not statistically significant, we obtained higher percentage of CpG methylation in certain tumors (Fig. 7A, B). Percent CpG methylation ranged from 3.13 to 12.3% in tumors, as compared to 3.03–4.64% in control.

For *Ret,* we analyzed a 468-bp fragment (37 CpGs, − 508 to − 56 relative to transcription start site, TSS). Similarly, there was no statistically significant differences in overall percent CpG methylation between tumor and control tissues. However, some tumors had a higher percentage of CpG methylation (Fig. 7A, B). Percent CpG methylation ranged from 2.95 to 13.78% in tumors, as compared to 2.7–4.05% in control. For *Nr4a1*, we analyzed a 500-bp fragment (47 CpGs, c.-530 to c.-89, relative to translation start site, ATG). In both tumor and control tissues, we observed very low methylation, averaging 2% (Fig. 7B). For *Fgfr4,* we analyzed a 391-bp fragment (19 CpGs, − 306 to 77, relative to transcription start site, TSS). Percent CpG methylation in both control and tumor tissues was 10% and below (Fig. 7B).

During bisulfite sequencing, we could also analyze for methylation at non-CpG sites (CpA, CpT, and CpC). Overall, there was no statistically significant differences in non-CpG methylation sites between tumor and control tissues (Fig. 7C). Nonetheless, in the promoter regions of *Nr4a1, Fgfr4* and *Ret,* we observed more tumors with higher number of non-CpG methylation sites than in control tissues (Fig. 7C). To summarize, bisulfite sequencing of the promoter regions of *Hrasls, Ret, Nr4a1* and *Fgfr4* showed a higher percentage of CpG methylation and a higher number of non-CpG methylation sites in specific tumors when compared to control tissues. There was also observed inter-tumor heterogeneity in the DNA methylation of promoter regions, irrespective of inducer or tumor type (Additional file 7: Figure S3A–C).

**Fig. 8:**
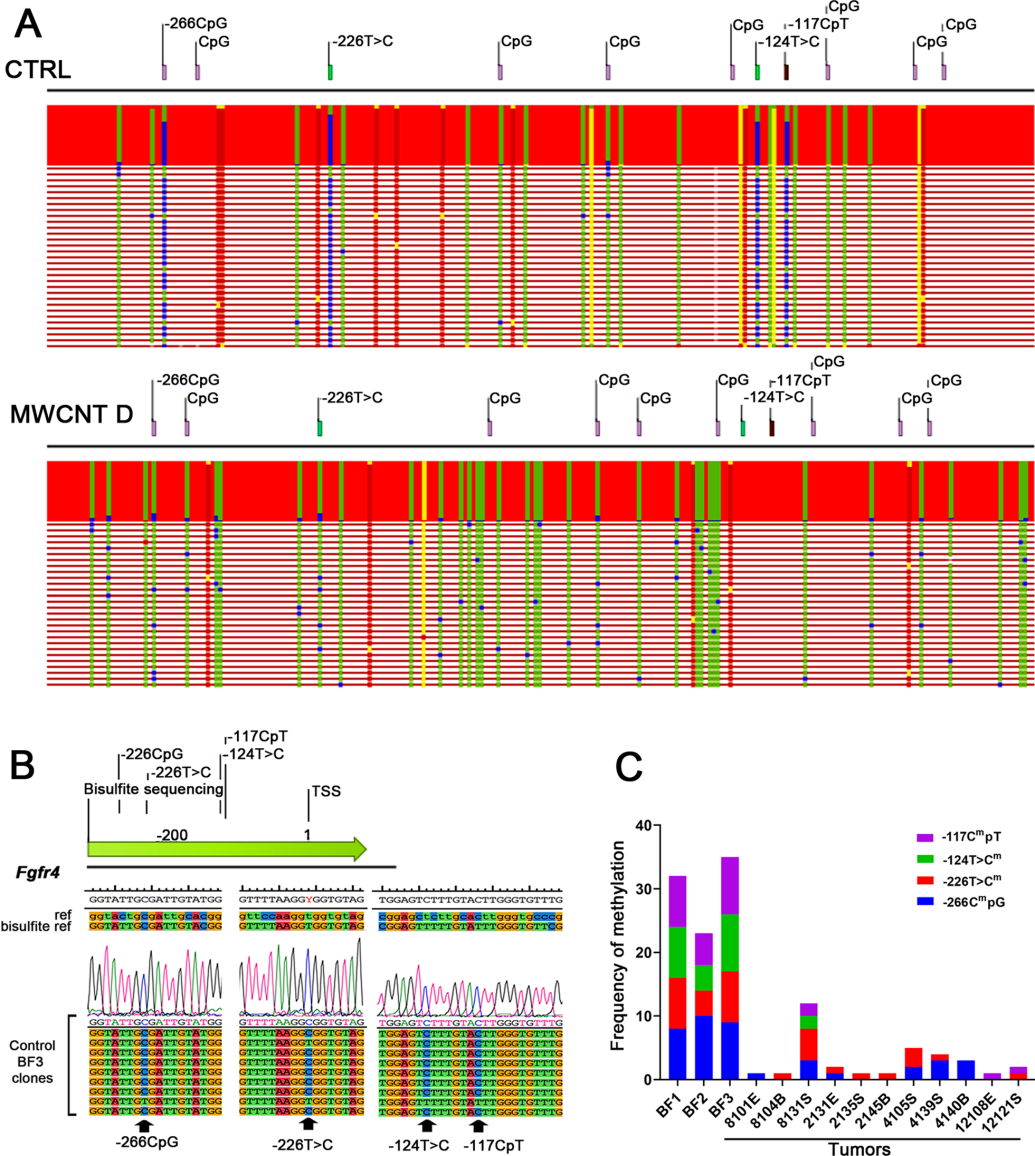
Hypomethylation of four sites in *Fgfr4* in tumors induced by MWCNTs and amosite asbestos. **A** Partial alignment of bisulfite sequences in control peritoneal tissues along the promoter region of *Fgfr4*, showing frequent methylation of all four sites: a CpG (− 266CpG), 2 sequence variations (− 226T > C; − 124T > C), and a non-CpG site (-117CpT). Methylated cytosines (blue boxes) are not converted into uracil by bisulfite treatment and remain unchanged. In contrast, similar alignment of bisulfite sequences in clones from tumors induced by MWCNT D did not show these frequently methylated sites. Instead, there is a high frequency of methylated cytosines in non-CpG sites. **B** Sequence electropherograms showing the four methylated sites in clones from a control peritoneal tissue (BF3). **C** Methylation frequency for the four sites in tumors and control tissues

Interestingly, along the promoter region of *Fgfr4* in control tissues, we identified four sites involving a CpG (− 266 CpG), 2 sequence variations (− 226 T > C; − 124 T > C), and a non-CpG (-117CpT) relative to TSS. We observed concurrent methylation of the cytosine of all four sites in 70% (21 of 30) of clones in control tissues, as compared to 0.9% (1 of 108) of clones in tumors (Fig. 8A–C). This result suggests hypomethylation of these sites in tumor tissues. Furthermore, we identified a sequence variation (-38A > G) that was present in 62% (67 of 108) of clones in tumor tissues. In contrast, this variation was present in only 3% (1 of 30) of clones in control tissues.

### Global DNA and RNA methylation levels are higher in tumors than in control tissues

**Fig. 9:**
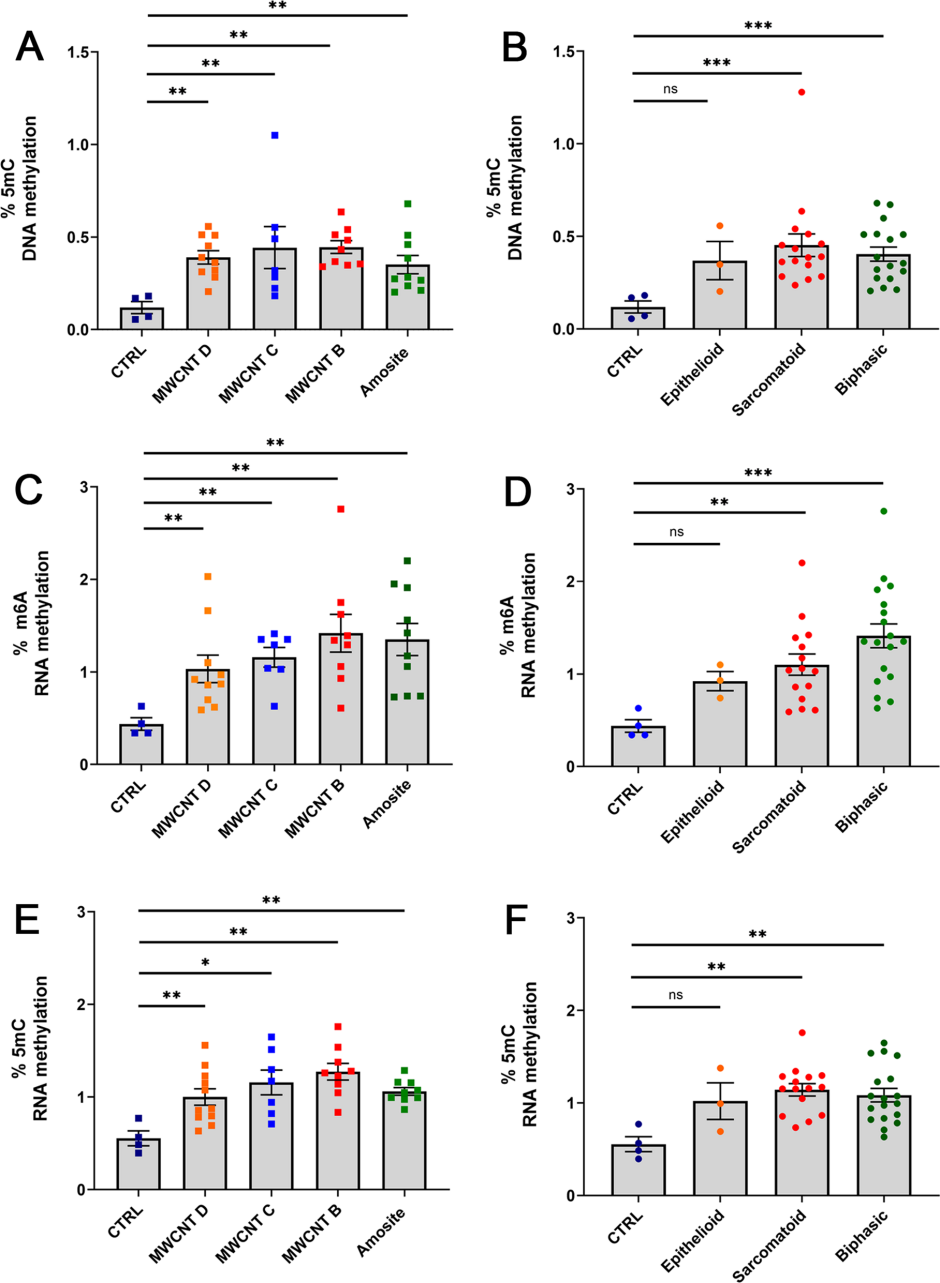
Global DNA and RNA methylation levels in tumors induced by MWCNTs or amosite asbestos. **A**, **B** Percent 5mC DNA methylation. **C**, **D** Percent m6A RNA methylation. **E**, **F** Percent 5mC RNA methylation. Results show global DNA and RNA methylation levels are higher in tumors than in control tissues. Scatter dot plots and statistical results were obtained using GraphPad Prism 9. Statistical significance was determined at *P* < 0.05, *t*–test for unpaired values, two-tailed. Bar depicts mean and standard error of the mean (SEM). Asterisks depict statistical significance at *** (*P* < 0. 001), **(*P* < 0. 01), * (*P* < 0. 05)

Local and global changes in methylation can lead to cancer development. These changes are not limited to DNA but can also occur in RNA and histone proteins [40]. To determine further the role of methylation in the development of the tumors after exposure to MWCNTs, we analyzed global DNA and RNA methylation levels in 36 tumors and 4 control peritoneal tissues (Fig. 9). We found that percent 5mC DNA, m6A RNA, and 5mC RNA methylation levels were overall higher in tumors, regardless of the inducer or tumor type, than in control peritoneal tissues. The presence of 5mC methylation in RNA was confirmed by performing bisulfite sequencing on 28S ribosomal RNA. This was undertaken in 35 clones from 5 MWCNT-induced rat tumors, and in 13 clones from human lung carcinoma A549 cells (see Section “Materials and Methods”).

## Discussion

So far, the development of malignant mesothelioma in patients is mostly attributed to asbestos exposure. Asbestos fibers can cause chronic inflammation over time, leading to immune activation, inflammatory microenvironment, and changes in the genome and epigenome conducive to malignant transformation [6]. Additionally, pathogenesis of mesothelioma can be driven by a dysregulated translatome, as shown by polysome profiling [41]. However, several studies have also demonstrated in animal models that exposure to certain carbon nanotubes (CNTs) can lead to adverse effects such as inflammation, fibrosis, and cancer, notably malignant mesothelioma [42]. Therefore, in this study, we aimed to gain further insights into the molecular cues of malignant mesothelioma development by multiwalled carbon nanotubes (MWCNTs). We investigated changes in the transcriptome and epigenome of tumors in Wistar rats, which were induced by intraperitoneal application of MWCNTs and amosite asbestos. We compared the tumors based on their inducer (MWCNTs or amosite asbestos) or their type (sarcomatoid, epithelioid or biphasic), to identify both common and distinct features.

Studies on animal models have shown that the route leading to malignancy, through chronic inflammation and molecular pathways of long-fiber CNTs, is identical to that of asbestos [22, 23]. We thus analyzed genome-wide transcriptome datasets from tumors induced by three different almost straight MWCNTs and amosite asbestos, to identify common malignancy features. Our analysis revealed several similarities between the transcriptome datasets of MWCNTs and amosite asbestos. For instance, the datasets had similar numbers of DEGs. Venn diagrams comparing the DEGs in the MWCNTs and amosite asbestos datasets showed that they shared an average of 70% of their DEGs. Among the commonly upregulated DEGs were *Msln* and *Spp1.* These genes encode for the proteins mesothelin and osteopontin (also known as secreted phosphoprotein 1), respectively. These proteins are also used for early diagnosis and prognosis for malignant pleural mesothelioma in patients [43].

Moreover, *Comparative Analysis* using Ingenuity Pathway Analysis revealed same top-scoring canonical pathways in the datasets of MWCNTs and amosite asbestos. Among these highly-scoring predicted activated canonical pathway were *IL8 Signaling* (Cytokine Signaling), and *Integrin Signaling* (Cell Cycle Regulation). IL8 (CXCL8) is a cytokine that causes inflammation. Signaling arises through binding of IL8 to one of two chemokine receptors, CXCR1 or CXCR2. IL8 expression can be regulated by exposures to environmental stressors to play an important role in inflammatory and cancer diseases [44]. Interestingly, IL8 has been shown as an autocrine growth factor for malignant mesothelioma [45]. Concerning *Integrin Signaling*, integrins are a family of cell surface receptors formed by combinations of α and β subunits. In mammals, there are 24 heterodimeric receptors that can bind to diverse ligands such as vitronectin, fibronectin and collagen, to mediate cell–cell and cell-extracellular matrix (ECM) interactions. The activation of integrin signaling is involved in inflammation and cancer [46, 47].

The *Comparative Analysis* also predicted same top scoring “*molecular function and diseases*” in the datasets of MWCNTs and amosite asbestos. These functions and diseases included *activation of cell movement*, *invasion of cells*, *migration of cells*, *cell transformation*, and *metastasis*. We also found 38 common DEGs implicated in mesothelioma or mesothelioma formation, which were either up- or downregulated across the transcriptome datasets of MWCNTs and amosite asbestos. While all these common features may be attributed to fiber morphology of the MWCNTs and amosite asbestos, we also found unique DEGs, which may be attributed to differences in fiber physico-chemical properties [17]. For instance, MWCNTs B, C, and D had diameters of 62 nm, 40 nm, and 37 nm, respectively. These MWCNTs were almost straight in their morphology. However, MWCNT B was the straightest, followed by MWCNT C, and then MWCNT D. Furthermore, the carbon source for MWCNT B and MWCNT C was cyclohexane, while that of MWCNT D was acetonitrile.

Indeed, the transcriptome datasets of MWCNT B and MWCNT C displayed more activated molecular functions than MWCNT D, which exhibited the smallest diameter and may have more flexibility. For example, we found that cancer-related functions including *neoplasia of cells, vasculogenesis,* or *growth of malignant tumor* (see Fig. 4A) were predicted to be activated in the datasets of MWCNT B and MWCNT C, but not MWCNT D. This increase in activated molecular functions seems to reflect and corroborate their tumor-inducing ability observed in the in vivo study [17]. In that study, the highest frequencies and earliest appearances of tumors were found with the rather straight MWCNTs such as MWCNT B. Additionally, in our efforts to characterize the same MWCNTs by using in vitro methods on primary human mesothelial (LP9) cells, we found also that the straighter MWCNTs mediated greater cytotoxicity and were more capable of inducing cellular senescence, compared to the less straight MWCNTs, and amosite asbestos [25]. For example, a markedly carcinogenic and straight MWCNT (MWCNT A/ MWCNT3) with a length of 8.57 µm, a diameter of 85 nm, and with benzene as carbon source exhibited extensive genome-wide changes in gene expression profile. Specifically, we identified 6110 DEGs for MWCNT A/ MWCNT3, 3376 for MWCNT D/ MWCNT1, and 499 for amosite asbestos.

It is known that the development of malignant mesothelioma in humans can span many years after exposure to carcinogenic fibers such as asbestos. In contrast, MWCNT-induced mesothelioma development in rats after intraperitoneal application has been shown to be very quick. However, the difference in lifespan between rats and humans must be considered. Despite this, the use of animal models is invaluable in understanding the molecular mechanisms of potential MWCNT-mediated carcinogenesis in humans. Therefore, we asked the question regarding the meaningful translation of findings obtained from rat tumors to human mesotheliomas. This study revealed, however, several probable avenues to pursue. For example, it would be interesting to investigate further the 38 DEGs identified here that are implicated in mesothelioma or mesothelioma formation. Of these 38 DEGs, 17 were similarly expressed in the datasets of human malignant pleural mesothelioma (GSE51024). Another example is the panel of six genes we validated by RT-qPCR in 36 tumors, which might serve as potential markers of certain types of mesotheliomas in patients. These genes were either downregulated (*Hrasls, Nr4a1, Fgfr4*, and *Ret*) or upregulated (*Rnd3* and *Gadd45b*) in all or most of the tumors relative to the control peritoneal tissues. Moreover, it is tempting to speculate about the role of *Gadd45b*, a gene implicated in carcinogenesis and identified by the *Causal Network Analysis* as a top master regulator.

Epigenetic mechanisms regulate gene activity and chromatin structure in normal mammalian cells. However, when these mechanisms are disrupted, they can lead to human diseases, including cancer [48]. Some well-known epigenetic mechanisms include DNA methylation, histone modifications, and non-coding RNAs. DNA methylation is a widely studied epigenetic mechanism of fundamental importance in mammalian development and disease. DNA can be modified by the covalent addition of a methyl group to position 5 of the cytosine ring, creating 5-methylcytosine. The addition of methyl group is catalyzed by enzymes called DNA methyltransferases (DNMTs). DNMT1 mediates the maintenance of DNA methylation, while DNMT3A and DNMT3B mediate de novo DNA methylation. In mammals, most methylation occurs at cytosines that are part of a C-G dinucleotide (known as CpGs). Methylation can also occur at non-CpG sites (CpA, CpT, and CpC), but its exact role in carcinogenesis is still unclear [49].

Aberrant DNA methylation patterns, either by hypermethylation or hypomethylation, are involved in cancer [50]. A recent systematic review and quantitative meta-analysis of DNA methylation in mesothelioma found both significantly hypomethylated genes (*APC*) and hypermethylated genes (*CDH1, ESR1, miR34b/c, PGR, RATO, SFRP1,* and *WIF1)* [51]. We investigated both global and gene-specific 5mC DNA methylation. Our data showed higher levels of global 5mC DNA methylation in rat tumors than control peritoneal tissues. To support this finding, our genome-wide transcriptome analysis showed a significant upregulation of DNA methylation maintenance *Dnmt1* (6–tenfold change) in tumors compared to control tissues. The analysis also revealed a significant 8–26-fold upregulation of *Uhrf1* (*Ubiquitin-like with PHD and ring finger domains 1*) in tumors compared to control. *UHRF1* and *DNMT1,* which are central regulators of DNA maintenance methylation, are often reported to be highly expressed in cancer cells [50]. In contrast, *Dnmt3* was downregulated, suggesting that no de novo DNA methylation was taking place in the tumors analyzed. Tumor heterogeneity has been reported in malignant pleural mesotheliomas with respect to transcriptomic, genetic, and epigenetic profiles [52–54]. Our DNA methylation analyses also revealed heterogeneity among the tumors. Bisulfite sequencing of *Hrasls, Ret, Nr4a1,* and *Fgfr4* revealed inter- and intra-tumor heterogeneity in the DNA methylation of promoter regions. We identified not only methylated CpGs, but also non-CpG methylated sites, and sequence variations. Interestingly, while certain tumors showed hypermethylation in the *Hrasls* promoter region, there was also evidence of hypomethylation in the *Fgfr4* promoter region.

Our data also showed higher levels of global m6A and 5mC RNA methylation in tumors compared to control peritoneal tissues. Indeed, misregulated RNA modifications and associated epitranscriptomic pathways (such as writers, erasers, and readers) have also been implicated in cancer [55]. One of the most well-known and abundant RNA mark is m6A, which is the methylation of adenosine at position 6 [56–58]. This modification is catalyzed by an RNA methyltransferase complex consisting of methyltransferase-like 3 (METTL3) and methyltransferase-like 14 (METTL14). It can be erased by m6A demethylases FTO or AlkB homolog 5 (ALKBH5) and interacts with m6A-binding proteins such as YTHDF1 and IGF2BP1. The methylation of carbon 5 in cytosine (5mC) in RNA has been discussed in recent reviews [59–61]. This modification has been detected in various RNA species, including ribosomal RNAs (rRNAs), transfer RNAs (tRNAs), messenger RNAs (mRNAs), enhancer RNAs (eRNAs), and non-coding RNAs. The process is catalyzed by enzymes from the NOL1/NOP2/SUN domain (NSUN) family, and the DNA methyltransferase-like 2 (DNMT2). 5mC plays a crucial role in regulating many aspects of gene expression such as by affecting RNA export, ribosome assembly, translation, and RNA stability.

We conducted a study to gain insights into disease development and molecular changes in tumors induced by exposure to asbestos or MWCNTs. Our findings could contribute to a better understanding of how exposure to MWCNTs can cause cancer. Such understanding is valuable for risk assessment and for the development of safe-by-design strategies to minimize potential risks associated with nanomaterials. For instance, we compared the transcriptome datasets of tumors induced by MWCNTs (differing in length, diameter, curvature, and carbon source), and amosite asbestos. Through this comparison, we were able to identify both common and distinct gene expression patterns, as well as affected molecular pathways. This information could be useful in identifying specific genes and molecular pathways associated with the development of malignant mesothelioma, and ultimately, in designing safer MWCNTs by correlating these findings with the physico-chemical characteristics of the different MWCNTs investigated. We also observed common activated molecular functions in the tumor datasets, such as *cell movement, invasion, migration, cell transformation, and metastasis*. This information could be added to the respective data pools to aid in designing MWCNTs that are less likely to induce these functions and promote tumor growth. Furthermore, the observation of expression changes in genes associated with malignant mesothelioma in humans within the rat tumor transcriptome datasets suggests similarities between rodent and human responses. This information could be used to extrapolate potential health effects of MWCNTs in humans and might add to respective safe-by-design strategies for these materials.

The mechanisms underlying mesothelioma pathogenesis after exposure to certain MWCNTs are still not well understood. Our study aimed to elucidate these mechanisms but had limitations due to its retrospective nature. We analyzed late-stage tumors, which may not provide a comprehensive understanding of the complex processes and factors involved. However, studying well-characterized tumors in animal models may still provide valuable insights into the molecular and cellular changes during carcinogenesis and contribute to a better understanding of specific mechanisms. Additionally, studying late-stage tumors may yield results better correlated with human mesotheliomas, which are usually detected at a late stage, potentially leading to the identification of therapeutic targets. Future research should analyze early-stage tumors which, however, may pose methodological challenges due to their more difficult detection, and the limited amount of cell material available for analysis. Moreover, these early-stage tumors should be compared with late-stage tumors. Another limitation is that we only analyzed changes in mRNA levels of genes implicated in cancer development using late-stage tumors. It is unclear if these changes truly reflect the process of carcinogenesis, although some here identified genes have been associated with carcinogenesis or malignant mesothelioma in patients. Further investigations and validation are needed to determine the relationship between these mRNA changes and the carcinogenicity caused by exposure to MWCNTs.

Altogether, we present findings that may help shed light on the disease development and its molecular signatures in tumors after exposure to asbestos or potentially carcinogenic MWCNTs. We analyzed malignant mesotheliomas induced by intraperitoneal application of MWCNTs and amosite asbestos in Wistar rats. Although the mesotheliomas induced by MWCNTs manifested earlier than those induced by amosite asbestos, they shared many similarities in their transcriptome and epigenome. Notably, they revealed many regulated genes similarly observed in human malignant mesotheliomas. Furthermore, global, and gene-specific methylation analyses of DNA and RNA showed epigenetic changes, although tumor heterogeneity was evident. In conclusion, mesothelioma pathogenesis from exposure to MWCNTs can likely result from the interplay of impaired pathways that impact the genome, epigenome and translatome, in combination with the immune response.

## Materials and methods

### MWCNTs and tissue materials

We analyzed tumor and control peritoneal tissues from Wistar rats, which were part of an in vivo study to investigate the carcinogenic effects of various MWCNTs. This in vivo study was reported previously by Rittinghausen et al. [17]. The procedures for intraperitoneal injection in rats, the morphology of MWCNTs and amosite asbestos, dosing schemes, and histological description of tumors were described in detail in that carcinogenicity study. Furthermore, the study was approved according to the German Animal Welfare Act by the local authority at the LAVES Niedersachsen, Hannover, Germany, No. 33.9-42502-04-11/0507; 33.9-42502-04-11/0743.

Briefly, a total of 500 (50 per group) male Wistar rats were treated intraperitoneally with four different tailor-made multiwalled carbon nanotubes (MWCNTs), and amosite asbestos. These MWCNTs (MWCNT A, MWCNT B, MWCNT C, and MWCNT D) differed in length, mesothelioma pathogenesis, diameter, and curvature. For example, the lengths of MWCNTs A–D were 8.57, 9.30, 10.24 and 7.91 µm, respectively. The diameters of MWCNTs A–D were 85, 62.25, 40.25 and 37.25 nm, respectively. Amosite asbestos had a length of 13.95 µm and a diameter of 394 nm. Additionally, the carbon source for MWCNT A was benzene, for MWCNTs B and C was cyclohexane, and for MWCNT D was acetonitrile. Further characterization of these MWCNTs was performed in another study using in vitro methods in primary human mesothelial LP9 cell [25]. In the Rittinghausen et al. study, all tested materials induced malignant mesotheliomas within two years. However, the highest frequencies and earliest appearances were observed with the straighter types, MWCNT A and B. Tumors were analyzed histologically and immuno-histochemically. The most frequent type of malignant mesothelioma was sarcomatoid (64%), followed by biphasic (32%). The epithelioid type was rare (4%). Malignant mesotheliomas of the sarcomatoid type were frequent in all MWCNT and amosite asbestos groups. Some resected tumors were flash-frozen in liquid nitrogen for further analysis. In the present study, we analyzed tumors induced by exposures to MWCNT B, MWCNT C, MWCNT D, and amosite asbestos (Additional file 4: Table S4). We also analyzed peritoneal tissues from the control group, which consisted of untreated rats from the Rittinghausen et al. study [17].

### Microarray analysis

Total RNA was isolated from tumors and control peritoneal tissues using the AllPrep DNA/RNA Mini Kit (Qiagen) according to the manufacturer’s protocol. For the microarray analysis, our initial objective was to identify differences in the transcriptome of tumors based on inducers. Therefore, we selected tumor samples primarily based on the inducers, and availability of sufficient frozen materials for downstream analysis. We then analyzed the data accordingly. Whenever possible, we included three different tumor types per inducer. We analyzed in total 11 tumors. Except for MWCNT B, we analyzed 3 tumors each for MWCNT C, MWCNT D, and amosite asbestos. Furthermore, we conducted data analysis based on tumor type to determine if the observations were influenced by the inducer or the specific subtype of mesothelioma. Of the 11 tumors, 3 were epithelioid, 5 were sarcomatoid, and 3 were biphasic, distributed randomly across the analyzed inducers. Additionally, we analyzed 3 control, tumor-free peritoneal tissues.

Genome-wide transcriptome analysis was undertaken using Affymetrix array (GeneChip® Rat Genome 230 2.0), and 3´IVT Plus Kit (Thermo Fisher Scientific). Sample preparation, labeling, hybridization, and quality control conditions were done according to manufacturer’s recommended protocols. Microarray data were subjected to quality control metrics contained in the Transcriptome Analysis Console Software (TAC 4.0.2, Thermo Fisher Scientific) and normalized using the PLIER method. Differential gene expression was determined using a filter criteria of fold change relative to control peritoneal tissue: > 2 or < − 2, ANOVA *P* < 0.05, and false discovery rate (FDR) *P* < 0.05.

We conducted additional bioinformatic analysis using Ingenuity Pathway Analysis (IPA, Qiagen). For each observation (inducer or tumor type), we uploaded the datasets that included all probe sets from the GeneChip® Rat Genome 230 2.0 into IPA. These datasets contained expression fold change, expression p-value, and expression false discovery rate, which were obtained using the TAC software. Next, we performed *Core Analysis* in IPA using the following criteria: fold change > 2 or < − 2, ANOVA *P* < 0.05, and false discovery rate (FDR) *P* < 0.05. The *Core Analysis* workflow generated results such as *Canonical Pathways, Upstream Regulators*, *Causal Networks*, as well as *Diseases and Functions*. Finally, we performed *Comparison Analysis* using the results from *Core Analysis* of each individual observation. In both analyses, we applied an activation z-score of at least > 2 or < − 2 to assess and explore various aspects, notably relevant predicted signaling pathways and biological functions. To compare our rat datasets with the datasets of human malignant pleural mesothelioma GSE51024 [27], and human small cell lung cancer (SCLC) tumor GSE149507 [28], we used the same bioinformatic tools and parameters.

### RT-qPCR

The mRNA expression of *Hrasls, Nr4a1, Fgfr4, Ret, Rnd3,* and *Gadd45b* was determined by RT-qPCR in 36 tumors and 4 control peritoneal tissues. The tumors were classified based on the type of inducer. There were 10 for MWCNT D, 7 for MWCNT C, 9 for MWCNT B, and 10 for amosite asbestos. In total, the tumors comprised 3 epithelioid, 15 sarcomatoid, and 18 biphasic mesotheliomas. Total RNA was isolated using the AllPrep DNA/RNA Mini Kit (Qiagen), according to the manufacturer’s recommendations. Reverse transcription of RNA was undertaken with the GrandScript cDNA Synthesis Supermix (TATAA). RT-qPCR was performed using standard procedures and conditions on a ViiA7 Real-Time PCR System (Thermo Fisher Scientific). Gene expression was determined by the delta-delta Ct method, with *Hprt1* gene expression as a reference. Statistically significant differences between tumors and control peritoneal tissues were determined by Student’s *t*–test for unpaired values (two-tailed) using GraphPad Prism 9. Statistical significance was determined at *P* < 0.05.

### Methylation analysis

Methylation analysis was undertaken on genomic DNA and RNA isolated from tumors and control peritoneal tissues using AllPrep DNA/RNA Kit (Qiagen), according to the manufacturer’s recommendations. Gene-specific methylation was determined by bisulfite sequencing in 11 tumors and 3 control peritoneal tissues. Bisulfite treatment of genomic DNA (1 µg) was performed using the EpiTect Bisulfite kit (Qiagen) according to the manufacturer’s instructions. Primers for amplification of fragments on bisulfite-treated DNA were designed using MethPrimer [62]. PCR fragments were either directly sequenced using the Sanger method on an ABI Prism 3130 XL Genetic Analyzer or cloned using the TOPO TA Cloning kit (Thermo Fisher Scientific) before sequencing. Sequences were analyzed using SeqMan Pro (Lasergene, DNASTAR). For each gene, we analyzed 8–10 clones from either control or tumor tissue to identify methylated sites. We then calculated the percent methylation by dividing the number of methylated CpGs by the total number of CpGs analyzed.

For bisulfite sequencing on 28S ribosomal RNA, we used total RNA from 5 rat MWCNT-induced tumors. Total RNA was bisulfite converted using EZ RNA Methylation Kit and bisulfite primers (Zymo). The bisulfite primers (H28SF/H28SR) amplified a 201 bp-fragment of human 28S ribosomal RNA (nucleotide position 4328–4528 GenBank NR_003287), which shares 100% homology to rat sequence. As a control, we also analyzed bisulfite converted RNA of human A549 cells. Bisulfite sequencing revealed methylation of 33 of 35 clones in tumors and 13 of 13 clones in A459 cells of cytosine at position 4447 (=rat 4184 NR_046246.2). We also observed additional methylated cytosines in tumors or in A549 cells in CpG and non-CpG sites.

For global DNA and RNA methylation analyses, we analyzed 36 tumors and 4 peritoneal tissues in a 96-well format. Global 5mC DNA methylation levels were detected using the 5mC DNA Methylation Colorimetric Assay Kit (Abcam ab233486) and recommended protocols. For the assay, we used 100 ng of input DNA from tumor or control tissue samples. Global m6A methylation levels were detected using the m6A RNA Methylation Colorimetric Assay Kit (Abcam ab185912) and recommended protocols. Global 5mC RNA methylation levels were detected with the 5mC RNA Methylation Fluorometric Assay Kit (5 Methyl Cytosine, Fluorometric, Abcam ab233492) and recommended protocols.

For both global 5mC and m6A RNA assays, we used 200 ng of input RNA from tumor or control tissue samples. Essentially, these global DNA and RNA methylation assays are based on quantifying the methylated fraction of bound nucleic acids on strip wells using capture and detection antibodies. The quantity of the methylated DNA fraction is determined by measuring absorbance at 450 nm (colorimetric) or fluorescence at Ex/Em = 530/590 nm (fluorometric in a microplate spectrophotometer. The percentage of methylated DNA or RNA is proportional to the OD intensity or fluorescence intensity measured.

### Supplementary Information

**Additional file 1**. Thirty-eight differentially expressed genes (DEGs), implicated in mesothelioma or its formation, which exhibited consistent expression changes across the transcriptome datasets of tumors classified by inducers.

**Additional file 2**. Thirty-eight differentially expressed genes (DEGs), implicated in mesothelioma or its formation, which were all up- or downregulated in the transcriptome datasets by tumor types or in the datasets of human malignant pleural mesotheliomas.

**Additional file 3**. List of 630 target genes of Gadd45b as a top master regulator identified in MWCNT B transcriptome dataset.

**Additional file 4**. Summary of analyzed tumors induced by MWCNTs or amosite asbestos, and control peritoneal tissues.

**Additional file 5.** Transcriptome profiling of tumors by mesothelioma types. (**A**) Venn diagrams display the quantity of genes that are common and unique among the datasets of sarcomatoid, biphasic, and epithelioid tumors. (**B**–**D**) Sample signals for the genes encoding forehead box M1 (Foxm1), mesothelin (Msln), and secreted phosphoprotein 1 (Spp1) in different tumor types and control peritoneal tissues. Samples (designated by dots) consisted of 5 sarcomatoid tumors, 3 biphasic tumors, 3 epithelioid tumors, and 3 control peritoneal tissues. Genes were filtered using fold change < − 2 or > 2, ANOVA *P* < 0.05, and FDR *P *< 0.05. Quality control criteria and bioinformatics tools were according to Transcriptome Analysis Console (TAC 4.0.2, Thermo Fisher Scientific).

**Additional file 6.** Genes implicated in mesothelioma or mesothelioma formation in rat tumors compared to human lung tumors. (**A**) Thirty-eight differentially expressed genes (DEGs) that showed consistent expression changes in all transcriptome datasets, regardless of inducer or tumor type. Overlay gene expressions (fold changes) represent those of MWCNT C. (**B**) The same set of genes is overlaid with gene expressions (fold changes) from the dataset GSE149507, which pertains to human small cell lung cancer (SCLC) tumors. Genes were filtered by fold change < − 1.5 or > 1.5, ANOVA P < 0.05, and FDR P < 0.05, and corresponding values are provided for each gene, respectively. Red (upregulated), green (downregulated), gray (did not meet at least one filter), orange (predicted activation).

**Additional file 7**. DNA methylation analysis in the promoter regions of *Hrasls, Ret, Nr4A1,* and *Fgfr4*, in tumors induced by MWCNTs or amosite asbestos, and control peritoneal tissues. (**A**) Percent CpG methylation by inducer. (**B**) Number of non-CpG methylation sites by inducer. (**C**) Percent CpG methylation by tumor type.

## Data Availability

The datasets generated and analyzed during the current study are available at Gene Expression Omnibus (GEO) repository, accession GSE233803.
