# Supplementary material for: Malignant peritoneal mesotheliomas of rats induced by multiwalled carbon nanotubes and amosite asbestos: transcriptome and epigenetic profiles
Source: Part Fibre Toxicol. 2024 Jan 31;21:3. doi: 10.1186/s12989-024-00565-x (PMC10829475; doi:10.1186/s12989-024-00565-x)
Supplement: Supplementary file 1 — Additional file 1. Thirty-eight differentially expressed genes (DEGs), implicated in mesothelioma or its formation, which exhibited consistent expression changes across the transcriptome datasets of tumors classified by inducers. [file 12989_2024_565_MOESM1_ESM.docx]

**Table S1-Thirty-eight differentially expressed genes (DEGs), implicated in mesothelioma or its formation, which exhibited consistent expression changes across the transcriptome datasets of tumors classified by inducers.**

| Genes |  | MWCNT B |  | MWCNT C |  | MWCNT D |  | Amosite |  |
| --- | --- | --- | --- | --- | --- | --- | --- | --- | --- |
|  |  | Fold Change | P-value | Fold Change | P-value | Fold Change | P-value | Fold Change | P-value |
| ADAM10 | ADAM metallopeptidase domain 10 | 12.15 | 4.13E-05 | 7.79 | 2.46E-05 | 11.57 | 5.97E-06 | 8.21 | 6.94E-05 |
| BCL10 | BCL10 immune signaling adaptor | 2.61 | 5.00E-04 | 2.56 | 2.00E-04 | 2.66 | 1.00E-04 | 2.07 | 2.30E-03 |
| CDH2 | cadherin 2 | 30.10 | 5.00E-03 | 23.83 | 5.50E-03 | 12.68 | 7.00E-03 | 21.02 | 1.70E-03 |
| DDX51 | DEAD-box helicase 51 | 3.29 | 3.00E-03 | 3.65 | 1.70E-03 | 5.50 | 2.00E-04 | 3.73 | 8.61E-05 |
| DHFR | dihydrofolate reductase | 6.51 | 9.85E-05 | 7.10 | 1.35E-05 | 3.76 | 6.00E-04 | 5.98 | 5.56E-05 |
| EP300 | E1A binding protein p300 | 3.12 | 1.00E-04 | 2.47 | 2.00E-04 | 3.04 | 5.38E-05 | 2.81 | 5.00E-04 |
| FLT3 | fms related receptor tyrosine kinase 3 | 13.64 | 5.00E-04 | 4.89 | 1.37E-02 | 21.17 | 6.30E-05 | 16.53 | 2.00E-04 |
| FOXM1 | forkhead box M1 | 37.02 | 3.47E-06 | 83.42 | 2.91E-07 | 16.54 | 5.18E-06 | 29.50 | 1.82E-06 |
| GLS | glutaminase | 2.63 | 2.00E-04 | 2.21 | 1.70E-03 | 2.03 | 1.50E-03 | 2.12 | 5.00E-04 |
| HDAC1 | histone deacetylase 1 | 9.83 | 2.19E-06 | 11.10 | 3.27E-07 | 8.54 | 1.32E-06 | 6.11 | 3.13E-06 |
| HSP90AA1 | heat shock protein 90 alpha family class A member 1 | 6.41 | 2.39E-06 | 6.82 | 5.69E-07 | 6.72 | 1.46E-06 | 6.87 | 6.72E-07 |
| HSP90B1 | heat shock protein 90 beta family member 1 | 5.10 | 1.91E-07 | 5.11 | 5.90E-08 | 4.71 | 1.02E-07 | 5.28 | 6.16E-08 |
| INHBA | inhibin subunit beta A | 55.62 | 5.00E-04 | 46.29 | 4.00E-04 | 19.26 | 4.50E-03 | 10.63 | 9.30E-03 |
| LYN | LYN proto-oncogene, Src family tyrosine kinase | 12.82 | 4.65E-05 | 12.05 | 1.85E-05 | 15.54 | 5.75E-06 | 11.72 | 3.31E-05 |
| MMUT | methylmalonyl-CoA mutase | -3.75 | 4.25E-06 | -3.06 | 2.98E-05 | -3.46 | 4.61E-06 | -2.78 | 8.86E-06 |
| MSLN | mesothelin | 172.40 | 4.00E-04 | 95.87 | 2.00E-04 | 282.06 | 5.00E-04 | 261.20 | 1.00E-04 |
| PIAS3 | protein inhibitor of activated STAT 3 | 2.77 | 7.00E-04 | 2.62 | 7.00E-04 | 2.27 | 1.50E-03 | 2.97 | 2.00E-04 |
| POR | cytochrome p450 oxidoreductase | 6.43 | 1.40E-05 | 5.47 | 9.01E-06 | 6.01 | 6.30E-06 | 6.27 | 7.67E-06 |
| PRR5 | proline rich 5 | 2.23 | 4.70E-03 | 2.09 | 7.50E-03 | 2.78 | 6.00E-04 | 3.28 | 1.10E-03 |
| RASSF1 | Ras association domain family member 1 | 3.16 | 8.99E-07 | 2.50 | 2.74E-06 | 3.11 | 3.59E-07 | 2.19 | 3.00E-04 |
| RRM2 | ribonucleotide reductase regulatory subunit M2 | 119.15 | 2.04E-06 | 116.94 | 7.45E-07 | 43.10 | 6.50E-06 | 51.39 | 3.96E-06 |
| SNAI1 | snail family transcriptional repressor 1 | 5.51 | 1.30E-03 | 4.77 | 4.00E-04 | 4.85 | 7.20E-03 | 2.95 | 1.47E-02 |
| SP1 | Sp1 transcription factor | 3.83 | 6.29E-06 | 4.15 | 8.52E-07 | 5.74 | 2.87E-07 | 3.56 | 3.86E-06 |
| SPP1 | secreted phosphoprotein 1 | 69.61 | 1.50E-03 | 78.64 | 6.00E-04 | 86.05 | 2.20E-03 | 42.04 | 2.30E-03 |
| SRC | SRC proto-oncogene, non-receptor tyrosine kinase | 23.41 | 2.00E-03 | 14.20 | 2.10E-03 | 22.31 | 7.00E-04 | 19.27 | 1.70E-03 |
| TOP2A | DNA topoisomerase II alpha | 26.48 | 2.09E-06 | 34.44 | 1.77E-06 | 17.41 | 2.57E-06 | 28.38 | 7.90E-07 |
| TOP2B | DNA topoisomerase II beta | 2.26 | 6.00E-04 | 2.48 | 2.00E-04 | 3.62 | 7.36E-06 | 2.30 | 7.22E-05 |
| TP53 | tumor protein p53 | 9.02 | 3.39E-07 | 11.62 | 3.31E-08 | 10.28 | 4.42E-08 | 9.07 | 5.71E-08 |
| TP63 | tumor protein p63 | -5.71 | 9.81E-05 | -4.36 | 2.00E-04 | -5.89 | 2.10E-05 | -4.26 | 6.24E-05 |
| TRAF7 | TNF receptor associated factor 7 | 2.53 | 7.95E-05 | 2.73 | 1.06E-05 | 2.62 | 3.22E-05 | 2.87 | 1.86E-05 |
| TUBA4A | tubulin alpha 4a | -17.26 | 3.51E-05 | -8.28 | 8.46E-05 | -4.91 | 1.50E-03 | -13.22 | 4.43E-05 |
| TUBA8 | tubulin alpha 8 | -185.95 | 3.99E-12 | -176.22 | 1.48E-12 | -188.97 | 1.20E-12 | -220.31 | 1.17E-12 |
| TUBB2A | tubulin beta 2A class IIa | 2.94 | 9.50E-03 | 2.94 | 5.30E-03 | 3.63 | 3.90E-03 | 4.55 | 2.50E-03 |
| TUBE1 | tubulin epsilon 1 | 2.77 | 1.20E-03 | 3.42 | 5.53E-05 | 2.49 | 1.10E-03 | 2.40 | 3.00E-04 |
| TYMS | thymidylate synthetase | 21.39 | 2.35E-05 | 18.93 | 5.31E-06 | 16.08 | 1.82E-05 | 19.14 | 7.82E-06 |
| ULK2 | unc-51 like autophagy activating kinase 2 | -2.88 | 4.00E-04 | -4.10 | 1.55E-05 | -2.69 | 6.78E-05 | -2.14 | 4.00E-04 |
| VIM | vimentin | 4.50 | 2.00E-04 | 4.31 | 1.00E-04 | 2.81 | 1.70E-03 | 4.08 | 6.00E-04 |
| WT1 | WT1 transcription factor | 316.88 | 7.11E-05 | 330.99 | 3.54E-05 | 177.52 | 2.00E-04 | 295.13 | 1.40E-05 |
