# Supplementary material for: Malignant peritoneal mesotheliomas of rats induced by multiwalled carbon nanotubes and amosite asbestos: transcriptome and epigenetic profiles
Source: Part Fibre Toxicol. 2024 Jan 31;21:3. doi: 10.1186/s12989-024-00565-x (PMC10829475; doi:10.1186/s12989-024-00565-x)
Supplement: Supplementary file 2 — Additional file 2. Thirty-eight differentially expressed genes (DEGs), implicated in mesothelioma or its formation, which were all up- or downregulated in the transcriptome datasets by tumor types or in the datasets of human malignant pleural mesotheliomas. [file 12989_2024_565_MOESM2_ESM.docx]

**Table S2-Thirty-eight differentially expressed genes (DEGs), implicated in mesothelioma or its formation, which were all up- or downregulated in the transcriptome datasets by tumor types or in the datasets of human malignant pleural mesotheliomas.**

| Genes |  | Sarcomatoid |  | Biphasic |  | Epithelioid |  | GSE51024, MPM |  |
| --- | --- | --- | --- | --- | --- | --- | --- | --- | --- |
|  |  | Fold Change | P-value | Fold Change | P-value | Fold Change | P-value | Fold Change | P-value |
| ADAM10 | ADAM metallopeptidase domain 10 | 8.16 | 2.50E-06 | 18.47 | 3.25E-07 | 13.22 | 8.04E-07 | 2.09 | 6.69E-07 |
| BCL10 | BCL10 immune signaling adaptor | 2.44 | 5.05E-05 | 2.56 | 3.00E-04 | 2.14 | 1.30E-03 | -1.33 | 3.01E-02 |
| CDH2 | cadherin 2 | 21.21 | 1.50E-03 | 36.64 | 1.10E-03 | 12.87 | 3.60E-03 | 4.61 | 1.72E-16 |
| DDX51 | DEAD-box helicase 51 | 3.34 | 4.00E-04 | 4.14 | 2.00E-04 | 3.70 | 6.94E-05 | 1.06 | 1.62E-01 |
| DHFR | dihydrofolate reductase | 6.57 | 2.33E-05 | 4.87 | 2.00E-04 | 4.78 | 4.00E-04 | 1.85 | 1.40E-14 |
| EP300 | E1A binding protein p300 | 2.94 | 7.99E-06 | 2.91 | 4.44E-05 | 2.30 | 3.00E-04 | -1.20 | 1.22E-02 |
| FLT3 | fms related receptor tyrosine kinase 3 | 6.16 | 1.60E-03 | 8.91 | 2.00E-03 | 23.28 | 1.00E-04 | 1.12 | 2.76E-02 |
| FOXM1 | forkhead box M1 | 49.02 | 4.05E-07 | 36.27 | 2.24E-06 | 28.87 | 7.88E-06 | 1.80 | 1.14E-12 |
| GLS | glutaminase | 2.11 | 1.00E-04 | 2.06 | 1.70E-03 | 2.51 | 5.37E-05 | 2.30 | 4.42E-06 |
| HDAC1 | histone deacetylase 1 | 10.48 | 1.16E-07 | 9.72 | 5.11E-07 | 6.04 | 2.28E-06 | -1.12 | 1.06E-02 |
| HSP90AA1 | heat shock protein 90 alpha family class A member 1 | 6.78 | 7.89E-08 | 6.98 | 8.59E-08 | 6.88 | 9.24E-08 | -1.08 | 1.60E-02 |
| HSP90B1 | heat shock protein 90 beta family member 1 | 4.96 | 4.43E-09 | 5.10 | 1.07E-08 | 5.73 | 6.64E-09 | 2.14 | 5.22E-08 |
| INHBA | inhibin subunit beta A | 34.77 | 2.10E-03 | 48.75 | 1.90E-03 | 18.77 | 6.60E-03 | 4.14 | 6.35E-07 |
| LYN | LYN proto-oncogene, Src family tyrosine kinase | 14.19 | 8.76E-07 | 14.39 | 3.61E-06 | 10.03 | 1.85E-05 | -1.31 | 1.20E-03 |
| MMUT | methylmalonyl-CoA mutase | -3.04 | 2.10E-06 | -3.18 | 3.13E-06 | -3.90 | 2.76E-07 | -1.18 | 4.22E-02 |
| MSLN | mesothelin | 91.76 | 1.35E-05 | 286.24 | 1.22E-06 | 286.49 | 1.24E-06 | 2.78 | 3.00E-04 |
| PIAS3 | protein inhibitor of activated STAT 3 | 2.47 | 2.00E-04 | 2.84 | 4.00E-04 | 3.14 | 9.90E-05 | -1.05 | 2.46E-01 |
| POR | cytochrome p450 oxidoreductase | 5.92 | 8.70E-07 | 6.33 | 1.74E-06 | 5.88 | 2.64E-06 | -1.39 | 1.00E-04 |
| PRR5 | proline rich 5 | 1.91 | 8.00E-04 | 3.46 | 2.88E-05 | 2.96 | 6.66E-05 | 1.83 | 2.60E-08 |
| RASSF1 | Ras association domain family member 1 | 2.63 | 1.73E-05 | 2.98 | 4.24E-05 | 3.04 | 6.15E-05 | -1.29 | 1.57E-02 |
| RRM2 | ribonucleotide reductase regulatory subunit M2 | 138.44 | 2.43E-07 | 61.20 | 2.30E-06 | 42.77 | 5.76E-06 | 5.51 | 2.16E-16 |
| SNAI1 | snail family transcriptional repressor 1 | 4.67 | 2.30E-03 | 4.50 | 5.30E-03 | 4.27 | 1.06E-02 | -1.08 | 6.34E-01 |
| SP1 | Sp1 transcription factor | 4.16 | 5.07E-07 | 4.10 | 6.45E-07 | 3.93 | 7.00E-04 | 1.14 | 1.11E-01 |
| SPP1 | secreted phosphoprotein 1 | 90.02 | 3.00E-04 | 67.15 | 5.00E-04 | 33.26 | 1.60E-03 | 30.84 | 1.85E-24 |
| SRC | SRC proto-oncogene, non-receptor tyrosine kinase | 18.26 | 5.00E-04 | 23.21 | 5.00E-04 | 22.16 | 6.00E-04 | -1.05 | 6.17E-01 |
| TOP2A | DNA topoisomerase II alpha | 31.03 | 2.52E-07 | 25.66 | 9.43E-07 | 21.42 | 1.92E-06 | 16.12 | 9.73E-27 |
| TOP2B | DNA topoisomerase II beta | 2.22 | 1.00E-04 | 2.80 | 9.14E-05 | 3.05 | 2.97E-05 | 1.13 | 6.61E-01 |
| TP53 | tumor protein p53 | 10.46 | 4.15E-09 | 10.45 | 2.49E-08 | 12.16 | 1.01E-08 | 1.91 | 9.97E-12 |
| TP63 | tumor protein p63 | -5.79 | 1.14E-05 | -5.49 | 8.36E-05 | -5.34 | 5.20E-05 | -1.54 | 3.25E-06 |
| TRAF7 | TNF receptor associated factor 7 | 2.68 | 1.85E-06 | 2.68 | 2.62E-06 | 2.71 | 1.47E-05 | 1.17 | 1.64E-05 |
| TUBA4A | tubulin alpha 4a | -12.79 | 2.14E-05 | -6.38 | 1.70E-03 | -10.56 | 9.87E-05 | -1.46 | 2.00E-04 |
| TUBA8 | tubulin alpha 8 | -161.44 | 8.53E-15 | -193.43 | 2.11E-14 | -214.31 | 2.13E-14 | 1.03 | 6.23E-01 |
| TUBB2A | tubulin beta 2A class IIa | 2.47 | 1.00E-03 | 4.36 | 7.71E-05 | 3.62 | 1.20E-03 | 2.62 | 5.24E-09 |
| TUBE1 | tubulin epsilon 1 | 2.46 | 1.00E-04 | 2.69 | 1.10E-03 | 3.25 | 2.00E-04 | -1.49 | 3.00E-03 |
| TYMS | thymidylate synthetase | 20.52 | 1.47E-06 | 19.79 | 2.13E-06 | 15.84 | 4.92E-06 | 4.01 | 3.54E-16 |
| ULK2 | unc-51 like autophagy activating kinase 2 | -3.08 | 5.45E-05 | -3.04 | 2.00E-04 | -3.02 | 2.00E-04 | 1.37 | 5.35E-05 |
| VIM | vimentin | 4.55 | 2.00E-04 | 4.12 | 5.00E-04 | 4.25 | 1.20E-03 | 1.53 | 8.16E-02 |
| WT1 | WT1 transcription factor | 282.52 | 1.83E-05 | 330.79 | 1.40E-05 | 433.53 | 4.03E-06 | 37.01 | 1.61E-21 |
