# Supplementary material for: Malignant peritoneal mesotheliomas of rats induced by multiwalled carbon nanotubes and amosite asbestos: transcriptome and epigenetic profiles
Source: Part Fibre Toxicol. 2024 Jan 31;21:3. doi: 10.1186/s12989-024-00565-x (PMC10829475; doi:10.1186/s12989-024-00565-x)
Supplement: Supplementary file 3 — Additional file 3. List of 630 target genes of Gadd45b as a top master regulator identified in MWCNT B transcriptome dataset. [file 12989_2024_565_MOESM3_ESM.docx]

**Table S3-List of 630 target genes of Gadd45b as a top master regulator identified in MWCNT B transcriptome dataset.**

| Target | Expr Fold Change | Molecule Type |
| --- | --- | --- |
| ABCA1 | 6.78 | transporter |
| ABCB1 | 19.34 | transporter |
| Abcb1b | 49.15 | transporter |
| ABCG1 | 9.89 | transporter |
| ACADL | -2.01 | enzyme |
| ACO2 | -5.05 | enzyme |
| ACSL5 | 6.01 | enzyme |
| ACSS1 | -12.64 | enzyme |
| ACTA1 | -216.25 | other |
| ACTA2 | -268.11 | other |
| ACTB | 3.49 | other |
| ACTC1 | -24.47 | enzyme |
| ACTN1 | 19.82 | transcription regulator |
| ACTN2 | -15018.90 | transcription regulator |
| Actn3 | -305.59 | other |
| ADAM10 | 12.15 | peptidase |
| ADAMTS9 | 2.04 | peptidase |
| ADCY3 | 5.24 | enzyme |
| ADGRG6 | 24.22 | G-protein coupled receptor |
| ADM | 8.48 | other |
| ADORA2B | 6.59 | G-protein coupled receptor |
| ADSS2 | 6.08 | enzyme |
| AHR | 6.10 | ligand-dependent nuclear receptor |
| AKT1 | 2.90 | kinase |
| ALAS1 | -3.33 | enzyme |
| ALDH1A3 | 20.44 | enzyme |
| ALDH7A1 | 3.18 | enzyme |
| ALDOC | 3.79 | enzyme |
| ANK1 | -427.74 | other |
| ANKRD1 | -2.03 | transcription regulator |
| ANXA5 | 6.92 | transporter |
| APOE | 8.11 | transporter |
| APP | 8.53 | other |
| AREG | 19.92 | growth factor |
| ARFGAP3 | 5.37 | transporter |
| ARL6 | 2.69 | transporter |
| ARPC2 | 3.75 | other |
| ARPP19 | 2.44 | transporter |
| ASF1A | -2.76 | other |
| ASF1B | 47.03 | other |
| ASPH | -381.31 | enzyme |
| ATAD2 | 7.75 | enzyme |
| ATF2 | 2.08 | transcription regulator |
| ATG7 | 2.70 | enzyme |
| ATP1A1 | 2.30 | transporter |
| ATP2A2 | -11.79 | transporter |
| ATP2B4 | 5.23 | transporter |
| ATP5F1A | -2.04 | transporter |
| Atp5k | -2.72 | enzyme |
| ATR | 7.80 | kinase |
| AURKA | 6.80 | kinase |
| AURKB | 30.92 | kinase |
| AXL | 3.48 | kinase |
| B3GNT5 | 43.00 | enzyme |
| B9D1 | 3.69 | other |
| BAD | 2.58 | other |
| BANP | -2.57 | other |
| BARD1 | 29.00 | transcription regulator |
| BBC3 | 5.90 | other |
| BCAR3 | 4.62 | other |
| BCKDHA | -3.26 | enzyme |
| BCL2A1 | 8.77 | other |
| BCL2L1 | 4.55 | other |
| BCL3 | 25.92 | transcription regulator |
| BGN | 10.34 | other |
| BHMT | 2.25 | enzyme |
| BLNK | 24.97 | other |
| BMF | 4.10 | other |
| BNIP3L | 2.09 | other |
| BRCA1 | 14.18 | transcription regulator |
| BRCA2 | 4.30 | transcription regulator |
| BTG3 | 8.63 | other |
| BUB1B | 21.57 | kinase |
| C1QA | 7.25 | other |
| C1QC | 10.24 | other |
| C1R | 7.27 | peptidase |
| C2 | 14.65 | peptidase |
| C3 | 63.98 | peptidase |
| C3AR1 | 15.33 | G-protein coupled receptor |
| CACNA1S | -880.37 | ion channel |
| CALB1 | 12.43 | other |
| CALR | 2.15 | transcription regulator |
| CAPN2 | 2.04 | peptidase |
| CAPN5 | 14.91 | peptidase |
| CASP1 | 3.89 | peptidase |
| CAV1 | 4.11 | transmembrane receptor |
| CAV3 | -820.08 | enzyme |
| CBR3 | 4.95 | enzyme |
| CBX5 | 2.56 | transcription regulator |
| Ccl2 | 9.54 | cytokine |
| CCNA1 | -97.42 | other |
| CCNB1 | 33.71 | kinase |
| CCNB2 | 26.36 | other |
| CCNE1 | 20.48 | transcription regulator |
| CCNE2 | 4.85 | other |
| CCNI | 2.53 | other |
| CCNL1 | 2.76 | other |
| CCNL2 | 2.99 | other |
| CCP110 | 2.70 | other |
| CCR5 | 22.42 | G-protein coupled receptor |
| CD14 | 9.75 | transmembrane receptor |
| CD44 | 14.63 | other |
| CD46 | 2.36 | transmembrane receptor |
| CD82 | 18.69 | other |
| CD83 | 5.94 | transmembrane receptor |
| CDC20 | 63.41 | other |
| CDC25B | 2.07 | phosphatase |
| CDC45 | 4.60 | other |
| CDCA3 | 28.18 | other |
| CDCA8 | 9.49 | other |
| CDH22 | -14.80 | other |
| CDH3 | 38.40 | other |
| CDK1 | 40.89 | kinase |
| CDK4 | 5.03 | kinase |
| CDKN1A | 4.23 | kinase |
| CDKN2C | 7.47 | transcription regulator |
| CDT1 | 9.15 | other |
| CEBPA | 2.35 | transcription regulator |
| CELSR1 | 75.44 | G-protein coupled receptor |
| CENPE | 155.46 | other |
| CENPM | 3.09 | other |
| CHCHD3 | -3.07 | transcription regulator |
| CHEK1 | 17.68 | kinase |
| CHEK2 | 10.63 | kinase |
| CHRNB1 | -38.16 | transmembrane receptor |
| CIRBP | 2.69 | translation regulator |
| CITED4 | -5.06 | transcription regulator |
| CKM | -334.95 | kinase |
| CKS2 | 36.89 | kinase |
| CLCF1 | 13.54 | cytokine |
| CLIP2 | 5.71 | transcription regulator |
| CNOT9 | 2.84 | transcription regulator |
| COL18A1 | 5.03 | other |
| COL5A2 | 6.23 | other |
| COPG1 | 2.73 | transporter |
| COQ5 | -2.19 | enzyme |
| COQ7 | -3.67 | enzyme |
| COX17 | -2.55 | enzyme |
| COX6A1 | 3.46 | enzyme |
| CP | 10.29 | enzyme |
| CPT1A | 12.84 | enzyme |
| CREM | -3.95 | transcription regulator |
| CRYAB | -9.52 | other |
| CSF1 | 6.55 | cytokine |
| CSF1R | 5.83 | kinase |
| CTSC | 2.35 | peptidase |
| CTTN | 19.76 | other |
| CXCL16 | 18.25 | cytokine |
| CXCL2 | 56.66 | cytokine |
| CXCL3 | 81.89 | cytokine |
| CXCL6 | 133.12 | cytokine |
| Cyb5r3 | 3.27 | enzyme |
| CYBB | 22.19 | enzyme |
| CYP3A4 | 4.18 | enzyme |
| CYSLTR1 | 2.08 | G-protein coupled receptor |
| DAB2 | 4.48 | other |
| DAP | 8.14 | transcription regulator |
| DDX39A | 3.03 | enzyme |
| DEGS1 | 3.96 | enzyme |
| DEK | 2.46 | transcription regulator |
| DEPDC1 | 37.54 | transcription regulator |
| DHRS1 | 2.86 | enzyme |
| DHX9 | 2.50 | enzyme |
| DIAPH3 | 70.66 | enzyme |
| DIO3 | 4.78 | enzyme |
| DLGAP5 | 17.37 | other |
| DMPK | -17.32 | kinase |
| DNAJB6 | -4.24 | transcription regulator |
| DNMT1 | 13.85 | enzyme |
| DNMT3A | -8.67 | enzyme |
| DSCC1 | 8.85 | other |
| DUSP5 | 6.56 | phosphatase |
| DUSP6 | 13.38 | phosphatase |
| DYNLT1 | 5.98 | other |
| E2F1 | 19.37 | transcription regulator |
| E2F7 | 14.64 | transcription regulator |
| E2F8 | 48.11 | transcription regulator |
| EDN1 | 4.48 | cytokine |
| EDNRA | 13.48 | transmembrane receptor |
| EFNB2 | 8.35 | kinase |
| EGR1 | 6.06 | transcription regulator |
| ELK1 | -2.54 | transcription regulator |
| ENO2 | 11.99 | enzyme |
| ENPP1 | 35.16 | enzyme |
| ENTPD4 | -3.09 | enzyme |
| EPHA2 | 20.68 | kinase |
| EPO | 2.25 | cytokine |
| ERAP1 | 3.23 | peptidase |
| ERO1A | 20.70 | enzyme |
| ESPL1 | 3.14 | peptidase |
| Esrra | -2.65 | transcription regulator |
| EZH2 | 13.54 | transcription regulator |
| EZR | 85.12 | other |
| F2RL1 | 8.84 | G-protein coupled receptor |
| F8 | 4.81 | other |
| FABP5 | 3.93 | transporter |
| FADD | 2.20 | other |
| FASN | -4.33 | enzyme |
| FBLN1 | 8.85 | other |
| FBXL12 | 2.98 | other |
| FCER1G | 11.40 | transmembrane receptor |
| FCGR3A/FCGR3B | 57.23 | transmembrane receptor |
| FDXR | 6.17 | enzyme |
| FGF21 | 3.86 | growth factor |
| FHL1 | -21.30 | other |
| FHL2 | 18.79 | transcription regulator |
| FLNA | 7.49 | other |
| FN1 | 6.98 | enzyme |
| FOSB | 2.25 | transcription regulator |
| FOSL1 | 25.86 | transcription regulator |
| FOSL2 | 4.95 | transcription regulator |
| FOXO1 | -6.00 | transcription regulator |
| FSCN1 | 15.62 | other |
| FTL | 5.97 | enzyme |
| FXYD1 | -15.99 | ion channel |
| FYB1 | 15.26 | other |
| FZD1 | 9.14 | G-protein coupled receptor |
| GAD2 | 4.24 | enzyme |
| GADD45GIP1 | -3.42 | other |
| GALR3 | 3.06 | G-protein coupled receptor |
| GAP43 | 8.52 | other |
| GAS5 | 2.22 | other |
| GAS6 | 4.62 | growth factor |
| GBP2 | 10.56 | enzyme |
| GCH1 | 37.86 | enzyme |
| GCLC | 7.48 | enzyme |
| GDF15 | 2.96 | growth factor |
| GDNF | 73.35 | growth factor |
| GINS1 | 8.87 | other |
| GINS3 | 3.11 | other |
| GINS4 | 4.58 | other |
| GJA1 | 74.60 | transporter |
| GLA | 2.81 | enzyme |
| GMNN | 23.74 | transcription regulator |
| GNAS | -3.44 | enzyme |
| GNPAT | -6.73 | enzyme |
| GOT1 | -3.07 | enzyme |
| GPR176 | 24.44 | G-protein coupled receptor |
| GPR27 | 7.44 | G-protein coupled receptor |
| GPR34 | 7.24 | G-protein coupled receptor |
| GPR61 | -4.43 | G-protein coupled receptor |
| GPR85 | 11.55 | G-protein coupled receptor |
| GPT2 | -9.10 | enzyme |
| GREM1 | 29.95 | other |
| GRTP1 | -11.69 | other |
| H2AX | 3.25 | transcription regulator |
| H3-3A/H3-3B | 5.48 | other |
| H4C11 | 2.75 | other |
| HAND2 | 61.18 | transcription regulator |
| HEY2 | 7.17 | transcription regulator |
| HIF1A | 2.90 | transcription regulator |
| HILPDA | 5.51 | other |
| HIP1 | 3.47 | other |
| HLA-A | 17.23 | other |
| HMGA1 | 10.64 | transcription regulator |
| HMGCR | 6.16 | enzyme |
| HMMR | 9.91 | transmembrane receptor |
| Hnrnpa3 | 3.49 | transporter |
| HOXA1 | -2.27 | transcription regulator |
| HPGD | 12.82 | enzyme |
| HRAS | -3.03 | enzyme |
| HSP90AA1 | 6.41 | enzyme |
| HSPA5 | 2.71 | enzyme |
| HUS1 | 2.50 | kinase |
| ICAM1 | 9.15 | transmembrane receptor |
| IFIH1 | 3.31 | enzyme |
| IFITM3 | 5.99 | other |
| IFNAR1 | 4.22 | transmembrane receptor |
| IFNK | -2.30 | cytokine |
| IGF2 | 3.59 | growth factor |
| IGFBP4 | 23.93 | other |
| IL10RA | 9.37 | transmembrane receptor |
| IL11 | 2.60 | cytokine |
| IL15 | -7.66 | cytokine |
| IL18 | 36.83 | cytokine |
| IL18R1 | 57.07 | transmembrane receptor |
| IL1B | 7.99 | cytokine |
| IL6 | 19.39 | cytokine |
| IL6ST | 2.65 | transmembrane receptor |
| IL7 | 5.35 | cytokine |
| INMT | 3.92 | enzyme |
| INPP1 | 2.55 | phosphatase |
| IRF1 | 4.15 | transcription regulator |
| IRF9 | 2.96 | transcription regulator |
| IRS1 | -4.60 | enzyme |
| ISL1 | 2.33 | transcription regulator |
| ITGAL | 5.40 | transmembrane receptor |
| ITGAM | 10.40 | transmembrane receptor |
| ITGAV | 18.60 | transmembrane receptor |
| ITGB1 | 3.18 | transmembrane receptor |
| ITGB2 | 15.25 | transmembrane receptor |
| ITGB8 | 39.49 | other |
| JAG2 | -3.17 | growth factor |
| JPT1 | 2.42 | other |
| JUND | 3.80 | transcription regulator |
| KDELR1 | 2.38 | transporter |
| KDELR2 | 3.74 | other |
| KDELR3 | 4.52 | transporter |
| KDM2A | 2.22 | enzyme |
| KDM5A | 3.19 | transcription regulator |
| KIF18B | 20.08 | other |
| KIF22 | 22.16 | other |
| KIF2C | 39.31 | other |
| KIF4A | 17.50 | other |
| KLF15 | -5.06 | transcription regulator |
| KLF3 | 3.97 | transcription regulator |
| KPNA2 | 4.38 | other |
| KRT17 | 761.97 | other |
| LAMB1 | 3.29 | other |
| LAMP2 | 4.37 | enzyme |
| LCP2 | 12.49 | other |
| LGMN | 4.65 | peptidase |
| LIG1 | 6.22 | enzyme |
| LMNB1 | 8.05 | other |
| LPIN1 | -8.26 | phosphatase |
| LRP1 | 3.20 | transmembrane receptor |
| LTBP3 | 3.63 | other |
| LUC7L | 2.23 | other |
| LYN | 12.82 | kinase |
| MAGED1 | 4.96 | transcription regulator |
| MAP2K6 | -14.71 | kinase |
| MAP2K7 | -2.34 | kinase |
| MAP4K4 | 7.51 | kinase |
| MAPK1 | -2.48 | kinase |
| MAPK3 | 9.20 | kinase |
| MAPK8 | -2.53 | kinase |
| MAPT | -39.93 | other |
| MATK | 3.41 | kinase |
| MBL2 | 6.60 | other |
| MCM10 | 15.67 | other |
| MCM2 | 9.07 | enzyme |
| MCM3 | 29.90 | enzyme |
| MCM4 | 5.41 | enzyme |
| MCM5 | 8.75 | enzyme |
| MCM6 | 27.19 | enzyme |
| MCM7 | 3.75 | enzyme |
| MDH1 | -3.72 | enzyme |
| MELK | 16.17 | kinase |
| MFHAS1 | 7.73 | other |
| MFN2 | -3.37 | enzyme |
| MIF | 4.30 | cytokine |
| MKI67 | 37.11 | other |
| MLYCD | -2.37 | enzyme |
| MMP11 | 17.55 | peptidase |
| MMP13 | 97.11 | peptidase |
| MMP14 | 8.88 | peptidase |
| MMS22L | 17.56 | other |
| MPC1 | -5.13 | transporter |
| MRPL11 | -2.81 | other |
| MRPL12 | -2.57 | other |
| MRPL53 | -2.02 | other |
| MRPL58 | -2.14 | enzyme |
| MRPS23 | -2.08 | other |
| MSH5 | 2.60 | enzyme |
| MSR1 | 7.90 | transmembrane receptor |
| MSTN | -423.39 | growth factor |
| MSTO1 | 2.16 | other |
| Mt1 | 71.53 | other |
| MTHFS | 2.18 | enzyme |
| MTSS1 | 10.18 | other |
| MUL1 | -2.52 | enzyme |
| MXD3 | 10.09 | transcription regulator |
| MYBL2 | 19.31 | transcription regulator |
| MYBPC1 | -3542.27 | other |
| MYBPH | -743.66 | other |
| MYH3 | -27.79 | enzyme |
| MYH4 | -5443.84 | enzyme |
| MYH8 | -2.08 | enzyme |
| MYL1 | -383.93 | other |
| MYLPF | -441.74 | other |
| MYOD1 | -212.66 | transcription regulator |
| MYOM2 | -927.81 | other |
| NASP | 4.40 | other |
| NCAPD2 | 6.77 | other |
| NCOA7 | 3.81 | transcription regulator |
| NDRG1 | 16.91 | kinase |
| NDRG4 | 3.87 | other |
| NDUFA10 | -3.64 | transporter |
| NDUFA13 | -2.49 | enzyme |
| NDUFA2 | -2.06 | enzyme |
| NDUFA3 | -2.89 | enzyme |
| NDUFA4 | -2.96 | enzyme |
| NDUFA5 | -2.93 | enzyme |
| NDUFA6 | -2.29 | enzyme |
| NDUFA9 | -4.32 | enzyme |
| NDUFB10 | -2.68 | enzyme |
| NDUFB8 | -3.60 | enzyme |
| NDUFS2 | -3.15 | enzyme |
| NDUFS7 | -4.00 | enzyme |
| NDUFV1 | -2.03 | enzyme |
| Nedd4 | 4.31 | enzyme |
| NFAT5 | 2.70 | transcription regulator |
| NFE2L1 | -2.10 | transcription regulator |
| NFE2L2 | 3.36 | transcription regulator |
| NFKB1 | 3.30 | transcription regulator |
| NFKB2 | 3.09 | transcription regulator |
| NFKBIA | 3.33 | transcription regulator |
| NFKBIZ | 8.75 | transcription regulator |
| NME1 | 4.23 | kinase |
| NOX1 | 3.00 | enzyme |
| NOX4 | 6.32 | enzyme |
| Nppb | 95.09 | other |
| NR4A1 | -15.33 | ligand-dependent nuclear receptor |
| NR4A2 | 2.96 | ligand-dependent nuclear receptor |
| NRF1 | 2.71 | transcription regulator |
| NSD3 | 2.47 | enzyme |
| NUAK2 | 10.79 | kinase |
| NUDT21 | 6.61 | other |
| NUMBL | 5.01 | other |
| NUP107 | 6.91 | other |
| NUP205 | 2.30 | other |
| NUPR1 | 5.45 | transcription regulator |
| OCLN | 5.89 | other |
| OLR1 | 183.95 | transmembrane receptor |
| OPRM1 | -2.07 | G-protein coupled receptor |
| ORC1 | 96.39 | other |
| ORC6 | 10.93 | other |
| P4HA2 | 4.67 | transporter |
| PA2G4 | 2.33 | transcription regulator |
| PARK7 | -2.66 | enzyme |
| PBX4 | 8.42 | transcription regulator |
| PCGF5 | -4.44 | other |
| PCLAF | 41.98 | other |
| PCTP | 3.02 | transporter |
| PDK4 | -8.49 | kinase |
| PDS5B | 2.20 | other |
| PEA15 | 6.00 | transporter |
| PENK | 22.10 | other |
| PER1 | 4.03 | transcription regulator |
| PEX13 | -2.12 | transporter |
| PGAM2 | -14630.62 | phosphatase |
| PGD | 5.18 | enzyme |
| PGP | -5.52 | enzyme |
| PKP1 | 1835.49 | other |
| PLD1 | 9.08 | enzyme |
| PLK1 | 41.58 | kinase |
| PLK2 | 5.04 | kinase |
| PLK3 | 7.82 | kinase |
| PLOD2 | 27.30 | enzyme |
| Pmaip1 | 19.50 | other |
| PML | 7.02 | transcription regulator |
| POLD1 | 3.71 | enzyme |
| POLE | 3.05 | enzyme |
| POU2F2 | 31.68 | transcription regulator |
| PPARGC1A | -84.68 | transcription regulator |
| PPIF | -2.71 | enzyme |
| PPM1D | 2.09 | phosphatase |
| PRDM1 | 4.01 | transcription regulator |
| PRDX1 | 2.40 | enzyme |
| PRDX4 | 6.14 | enzyme |
| PRIM2 | 13.13 | enzyme |
| PRKCD | 7.92 | kinase |
| PRMT1 | 2.21 | enzyme |
| PSEN2 | 2.23 | peptidase |
| PSIP1 | 3.83 | transcription regulator |
| PSMB9 | 11.95 | peptidase |
| PTGFR | 56.82 | G-protein coupled receptor |
| PTTG1 | 32.04 | transcription regulator |
| Pvr | 4.22 | other |
| PYGL | 3.96 | enzyme |
| RAC2 | 6.45 | enzyme |
| RACGAP1 | 30.34 | transporter |
| RAD18 | 7.52 | other |
| RAD21 | 2.31 | transcription regulator |
| RAD51 | 18.47 | enzyme |
| RAD51AP1 | 2.96 | other |
| RAMP3 | 15.36 | G-protein coupled receptor |
| RAN | 2.16 | enzyme |
| RANBP1 | 5.72 | other |
| RARA | 2.22 | ligand-dependent nuclear receptor |
| RASSF1 | 3.16 | other |
| RBL1 | 10.57 | transcription regulator |
| RBP1 | 127.02 | transporter |
| RBPJ | 4.64 | transcription regulator |
| RCN3 | 4.83 | other |
| REEP1 | -9.13 | other |
| RELA | 2.13 | transcription regulator |
| Retnla | 2.02 | other |
| RFC1 | 3.31 | transcription regulator |
| RFC2 | 2.28 | enzyme |
| RFC3 | 3.51 | enzyme |
| RHOB | 2.51 | enzyme |
| RNF138 | 2.26 | enzyme |
| ROCK1 | 3.55 | kinase |
| RORA | -5.40 | ligand-dependent nuclear receptor |
| RRAD | -33.58 | enzyme |
| RRAGD | -47.73 | enzyme |
| RRM1 | 2.58 | enzyme |
| RRM2 | 119.15 | enzyme |
| RT1-EC2 | 14.23 | other |
| SART3 | 2.45 | other |
| SAT1 | 5.77 | enzyme |
| SATB2 | 29.24 | transcription regulator |
| SCD | -97.86 | enzyme |
| SDC1 | 1018.83 | enzyme |
| SDC2 | 6.51 | other |
| SDC4 | 8.38 | other |
| SDHB | -2.77 | enzyme |
| SDHD | -3.21 | enzyme |
| SEC11A | 3.24 | peptidase |
| SENP1 | 2.29 | peptidase |
| SENP2 | -2.24 | peptidase |
| SEPTIN3 | 5.38 | enzyme |
| SERP1 | 7.96 | other |
| SERPINB1 | 6.08 | other |
| SERTAD1 | 2.06 | transcription regulator |
| SETD5 | 2.72 | enzyme |
| SF3B1 | 3.61 | other |
| SFTPA1 | 2.50 | transporter |
| SFTPB | 2.65 | other |
| SH3BP4 | 7.66 | other |
| SH3GLB1 | -5.74 | enzyme |
| SH3RF1 | 7.17 | enzyme |
| SHC1 | 2.92 | other |
| SHH | -4.02 | peptidase |
| SLBP | -7.15 | other |
| SLC12A7 | 3.54 | transporter |
| SLC2A1 | 9.25 | transporter |
| SLC2A2 | 3.23 | transporter |
| SLC2A3 | 16.11 | transporter |
| SLC2A4 | -118.53 | transporter |
| SLC37A4 | -4.86 | transporter |
| SLC3A1 | -2.98 | transporter |
| SLC4A7 | 6.05 | transporter |
| SLC6A2 | -3.40 | transporter |
| SLC7A5 | 19.17 | transporter |
| Slfn2 | 4.32 | other |
| SLIT2 | 116.46 | other |
| SMAD7 | 4.29 | transcription regulator |
| SMC1A | 2.21 | transporter |
| SMC3 | 2.21 | other |
| SMC4 | 5.82 | transporter |
| SMURF2 | 5.48 | enzyme |
| SNAI1 | 5.51 | transcription regulator |
| SOST | 3.66 | other |
| SOX2 | 2.60 | transcription regulator |
| SOX4 | 23.89 | transcription regulator |
| SOX5 | 11.59 | transcription regulator |
| SPAG5 | 31.07 | peptidase |
| SPC24 | 26.24 | other |
| SPC25 | 21.43 | other |
| SPHK1 | 17.52 | kinase |
| SPTBN1 | 4.22 | other |
| SRPK1 | 2.16 | kinase |
| SRSF2 | 2.44 | transcription regulator |
| SSRP1 | 3.34 | transcription regulator |
| ST18 | 2.79 | transcription regulator |
| STAR | 17.73 | transporter |
| STAT1 | 2.95 | transcription regulator |
| STAT3 | 3.04 | transcription regulator |
| STC2 | 6.51 | other |
| STIM1 | -3.94 | ion channel |
| STK10 | 4.73 | kinase |
| STK32C | 5.84 | kinase |
| STMN1 | 10.31 | other |
| SUPT3H | 2.29 | transcription regulator |
| SUV39H1 | 3.45 | enzyme |
| TACC3 | 21.79 | other |
| TAP1 | 16.30 | transporter |
| TAP2 | 8.08 | transporter |
| TBP | 2.10 | transcription regulator |
| TCF19 | 13.46 | transcription regulator |
| TENT5A | 6.45 | other |
| TFRC | -7.59 | transporter |
| TGFA | 121.33 | growth factor |
| TGFB1 | 9.06 | growth factor |
| TIMELESS | 10.64 | other |
| TIMP1 | 44.83 | cytokine |
| TK1 | 17.31 | kinase |
| TLR4 | 4.25 | transmembrane receptor |
| TLR8 | 10.09 | transmembrane receptor |
| TMEM176A | 6.02 | other |
| TMEM176B | 7.08 | other |
| TMEM50B | -3.70 | other |
| TMOD2 | -2.97 | other |
| TMPO | 5.02 | other |
| TNFAIP2 | 7.46 | other |
| TNFAIP8 | 9.10 | other |
| TNFRSF1A | 3.09 | transmembrane receptor |
| TNFRSF1B | 16.35 | transmembrane receptor |
| TNFRSF9 | 3.76 | transmembrane receptor |
| TNFSF11 | 34.01 | cytokine |
| TNN | 81.62 | other |
| TNNI1 | -132.72 | other |
| TNNI2 | -72.09 | enzyme |
| TNNT1 | -131.11 | other |
| TOPBP1 | 5.24 | other |
| TPD52 | 3.80 | other |
| Tpm3 | -74.42 | other |
| TRADD | 4.88 | other |
| TRAF1 | 14.54 | other |
| TRH | 57.68 | other |
| TRIB3 | 10.91 | kinase |
| Trim30a/Trim30d | 9.57 | other |
| TRIM72 | -414.78 | enzyme |
| TRIP13 | 26.05 | transcription regulator |
| TRPC6 | 46.38 | ion channel |
| TSLP | 4.27 | cytokine |
| TUBB | 13.24 | other |
| TUBB2B | 3.64 | other |
| TXN2 | -2.39 | enzyme |
| TYMS | 21.39 | enzyme |
| UBR2 | -2.03 | enzyme |
| UGCG | 2.78 | enzyme |
| UGT1A6 | 6.55 | enzyme |
| ULK1 | -2.05 | kinase |
| UNG | 7.57 | enzyme |
| UQCRFS1 | -2.08 | enzyme |
| USP1 | 7.24 | peptidase |
| VASP | 3.74 | other |
| VCAM1 | 43.57 | transmembrane receptor |
| VCAN | 164.84 | other |
| VDR | 10.68 | transcription regulator |
| VIM | 4.50 | other |
| VLDLR | -8.71 | transporter |
| VSIR | 7.54 | other |
| WDR90 | 3.82 | other |
| WEE1 | 5.40 | kinase |
| WNT10A | 11.10 | other |
| WT1 | 316.88 | transcription regulator |
| XDH | 3.24 | enzyme |
| XIAP | 2.67 | enzyme |
| XPO1 | 2.12 | transporter |
| ZC3H12A | -2.61 | enzyme |
| ZEB2 | 2.78 | transcription regulator |
| ZFP36 | 2.50 | transcription regulator |
| ZFP36L1 | 3.46 | transcription regulator |
| ZKSCAN1 | 2.93 | transcription regulator |
| ZMAT3 | 2.84 | other |
| ZYX | 2.40 | other |
