# Supplementary material for: Malignant peritoneal mesotheliomas of rats induced by multiwalled carbon nanotubes and amosite asbestos: transcriptome and epigenetic profiles
Source: Part Fibre Toxicol. 2024 Jan 31;21:3. doi: 10.1186/s12989-024-00565-x (PMC10829475; doi:10.1186/s12989-024-00565-x)
Supplement: Supplementary file 4 — Additional file 4. Summary of analyzed tumors induced by MWCNTs or amosite asbestos, and control peritoneal tissues. [file 12989_2024_565_MOESM4_ESM.docx]

**Table S4-Summary of analyzed tumors induced by MWCNTs or amosite asbestos, and control peritoneal tissues.**

| Material  ID | Inducer | Tumor type | Affymetrix  microarray | RT-qPCR | Bisulfite sequencing  DNA methylation | Global DNA 5mC methylation | Global RNA m6A methylation | Global RNA 5mC methylation |
| --- | --- | --- | --- | --- | --- | --- | --- | --- |
| 8101 | Amosite | Epithelioid | x | x | x | x | x | x |
| 8114 | Amosite | Sarcomatoid | - | x | - | x | x | x |
| 8120 | Amosite | Sarcomatoid | - | x | - | x | x | - |
| 8127 | Amosite | Sarcomatoid | - | x | - | x | x | x |
| 8131 | Amosite | Sarcomatoid | x | x | x | x | x | x |
| 8144 | Amosite | Sarcomatoid | - | x | - | x | x | x |
| 8104 | Amosite | Biphasic | x | x | x | x | x | x |
| 8119 | Amosite | Biphasic | - | x | - | x | x | x |
| 8139 | Amosite | Biphasic | - | x | - | x | x | x |
| 8136 | Amosite | Biphasic | - | x | - | x | x | x |
| 2131 | MWCNT D | Epithelioid | x | x | x | x | x | x |
| 2135 | MWCNT D | Sarcomatoid | x | x | x | x | x | x |
| 2112 | MWCNT D | Biphasic | - | x | - | x | x | x |
| 2116 | MWCNT D | Biphasic | - | x | - | x | x | x |
| 2125 | MWCNT D | Biphasic | - | x | - | x | x | x |
| 2145 | MWCNT D | Biphasic | x | x | x | x | x | x |
| 3112 | MWCNT D | Sarcomatoid | - | x | - | x | x | x |
| 3138 | MWCNT D | Sarcomatoid | - | x | - | x | x | x |
| 3148 | MWCNT D | Sarcomatoid | - | x | - | x | x | x |
| 3131 | MWCNT D | Biphasic | - | x | - | x | x | x |
| 3123 | MWCNT D | Sarcomatoid |  |  |  | x |  | x |
| 4105 | MWCNT C | Sarcomatoid | x | x | x | x | x | x |
| 4139 | MWCNT C | Sarcomatoid | x | x | x | x | x | x |
| 4111 | MWCNT C | Biphasic | - | x | - | x | x | x |
| 4118 | MWCNT C | Biphasic | - | x | - | x | x | x |
| 4138 | MWCNT C | Biphasic | - | x | - | x | x | x |
| 4140 | MWCNT C | Biphasic | x | x | x | x | x | x |
| 4144 | MWCNT C | Biphasic | - | x | - | x | x | x |
| 11107 | MWCNT B | Biphasic | - | x | - | x | x | x |
| 11135 | MWCNT B | Biphasic | - | x | - | x | x | x |
| 11146 | MWCNT B | Biphasic | - | x | - | x | x | x |
| 12107 | MWCNT B | Sarcomatoid | - | x | - | x | x | x |
| 12110 | MWCNT B | Sarcomatoid | - | x | - | x | x | x |
| 12121 | MWCNT B | Sarcomatoid | x | x | x | x | x | x |
| 12141 | MWCNT B | Sarcomatoid | - | x | - | x | x | - |
| 12108 | MWCNT B | Epithelioid | x | x | x | x | x | x |
| 12116 | MWCNT B | Biphasic | - | x | - | x | x | x |
| BF1 | - | Ctrl | x | x | x | x | x | x |
| BF2 | - | Ctrl | x | x | x | x | x | x |
| BF3 | - | Ctrl | x | x | x |  | x | x |
| BF4 | - | Ctrl | - | x | - | x | x | x |
| BF5 | - | Ctrl |  |  |  | x |  |  |
