# Supplementary material for: Malignant peritoneal mesotheliomas of rats induced by multiwalled carbon nanotubes and amosite asbestos: transcriptome and epigenetic profiles
Source: Part Fibre Toxicol. 2024 Jan 31;21:3. doi: 10.1186/s12989-024-00565-x (PMC10829475; doi:10.1186/s12989-024-00565-x)
Supplement: Supplementary file 5 — Additional file 5. Transcriptome profiling of tumors by mesothelioma types. (A) Venn diagrams display the quantity of genes that are common and unique among the datasets of sarcomatoid, biphasic, and epithelioid tumors. (B–D) Sample signals for the genes encoding forehead box M1 (Foxm1), mesothelin (Msln), and secreted phosphoprotein 1 (Spp1) in different tumor types and control peritoneal tissues. Samples (designated by dots) consisted of 5 sarcomatoid tumors, 3 biphasic tumors, 3 epithelioid tumors, and 3 control peritoneal tissues. Genes were filtered using fold change < − 2 or > 2, ANOVA P < 0.05, and FDR P < 0.05. Quality control criteria and bioinformatics tools were according to Transcriptome Analysis Console (TAC 4.0.2, Thermo Fisher Scientific). [file 12989_2024_565_MOESM5_ESM.pdf]

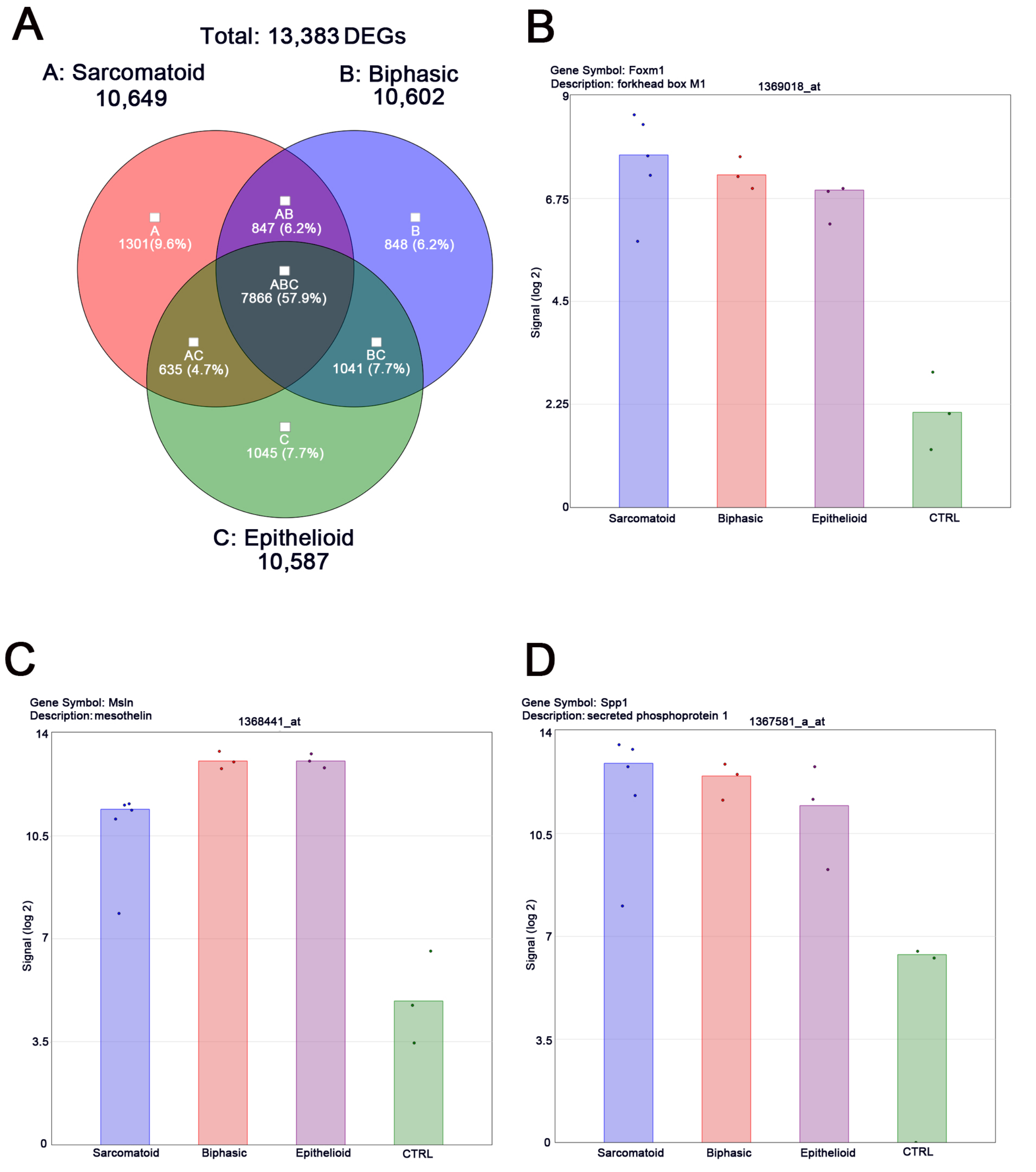
A
Total: 13,383 DEGs
A: Sarcomatoid
10,649
B: Biphasic
10,602
A
1301(9.6%)
AB
847 (6.2%)
B
848 (6.2%)
ABC
7866 (57.9%)
AC
635 (4.7%)
BC
1041 (7.7%)
C
1045 (7.7%)
C: Epithelioid
10,587
B
Gene Symbol: Foxm1
Description: forkhead box M1
1369018_at
Signal (log 2)
9
6.75
4.5
2.25
0
Sarcomatoid
Biphasic
Epithelioid
CTRL
C
Gene Symbol: Msln
Description: mesothelin
1368441_at
Signal (log 2)
14
10.5
7
3.5
0
Sarcomatoid
Biphasic
Epithelioid
CTRL
D
Gene Symbol: Spp1
Description: secreted phosphoprotein 1
1367581_a_at
Signal (log 2)
14
10.5
7
3.5
0
Sarcomatoid
Biphasic
Epithelioid
CTRL
