# Supplementary figures and images for: Malignant peritoneal mesotheliomas of rats induced by multiwalled carbon nanotubes and amosite asbestos: transcriptome and epigenetic profiles
Source: Part Fibre Toxicol. 2024 Jan 31;21:3. doi: 10.1186/s12989-024-00565-x (PMC10829475; doi:10.1186/s12989-024-00565-x)

# A rat mesothelioma

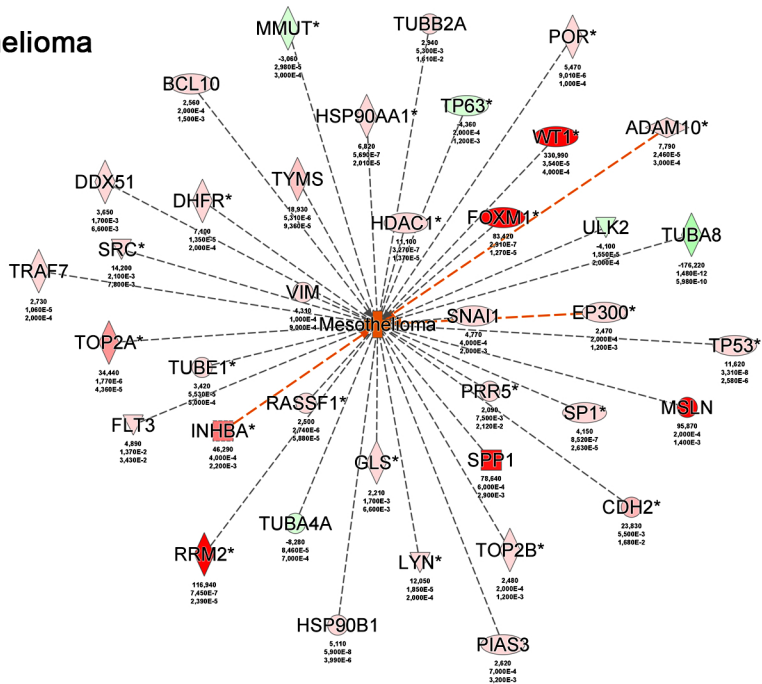

# B human lung tumor (SCLC)

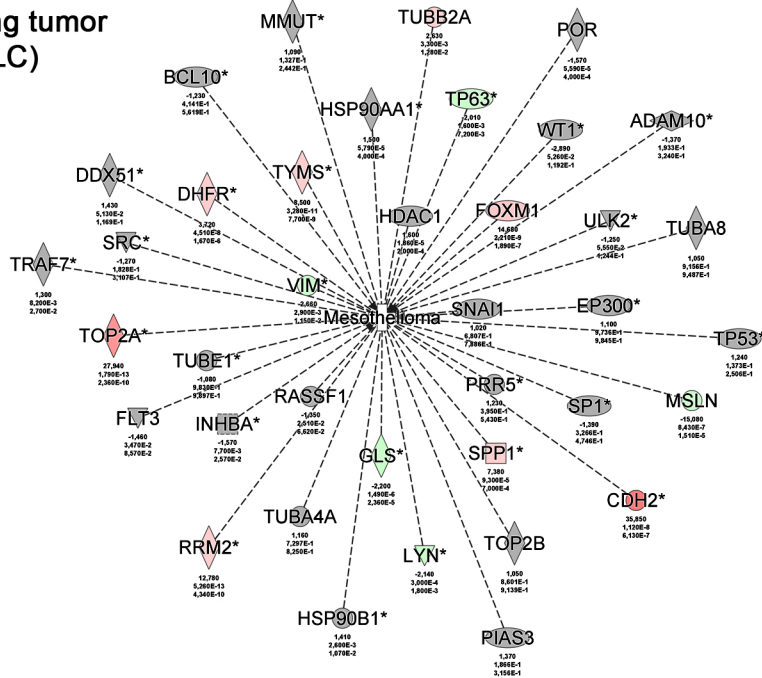

Supplement: Supplementary file 6 — Additional file 6. Genes implicated in mesothelioma or mesothelioma formation in rat tumors compared to human lung tumors. (A) Thirty-eight differentially expressed genes (DEGs) that showed consistent expression changes in all transcriptome datasets, regardless of inducer or tumor type. Overlay gene expressions (fold changes) represent those of MWCNT C. (B) The same set of genes is overlaid with gene expressions (fold changes) from the dataset GSE149507, which pertains to human small cell lung cancer (SCLC) tumors. Genes were filtered by fold change < − 1.5 or > 1.5, ANOVA P < 0.05, and FDR P < 0.05, and corresponding values are provided for each gene, respectively. Red (upregulated), green (downregulated), gray (did not meet at least one filter), orange (predicted activation). [file 12989_2024_565_MOESM6_ESM.pdf]

**A**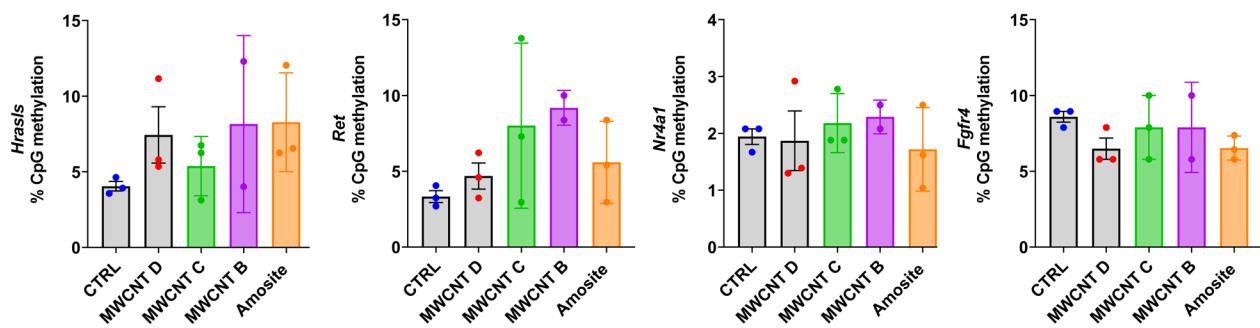**B**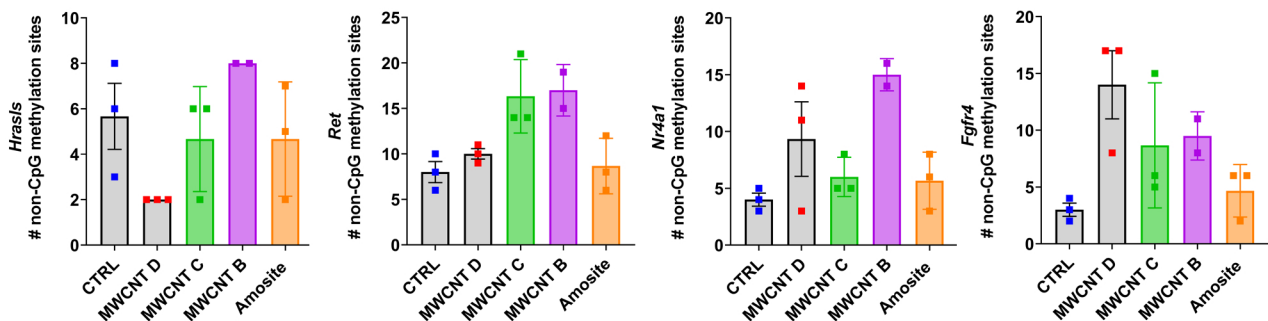**C**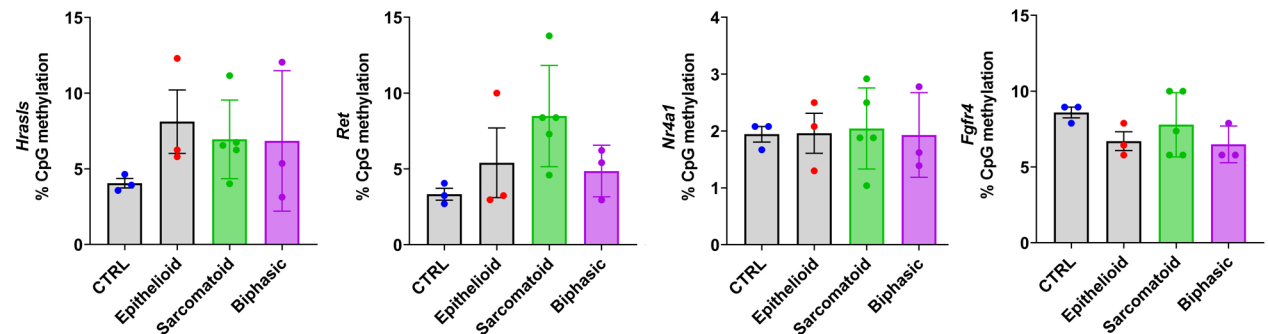

Supplement: Supplementary file 7 — Additional file 7. DNA methylation analysis in the promoter regions of Hrasls, Ret, Nr4A1, and Fgfr4, in tumors induced by MWCNTs or amosite asbestos, and control peritoneal tissues. (A) Percent CpG methylation by inducer. (B) Number of non-CpG methylation sites by inducer. (C) Percent CpG methylation by tumor type. [file 12989_2024_565_MOESM7_ESM.pdf]
